# Characterization of a novel 4-guanidinobutyrase from *Candida parapsilosis*

**DOI:** 10.1093/femsyr/foae003

**Published:** 2024-01-18

**Authors:** Santoshkumar R Gaikwad, Narayan S Punekar, Ejaj K Pathan

**Affiliations:** Molecular Enzymology Laboratory, Department of Biosciences and Bioengineering, Indian Institute of Technology Bombay, Powai, Mumbai 400076, Maharashtra, India; Molecular Enzymology Laboratory, Department of Biosciences and Bioengineering, Indian Institute of Technology Bombay, Powai, Mumbai 400076, Maharashtra, India; Department of Biosciences and Bioengineering, Indian Institute of Technology Dharwad, Dharwad 580011, Karnataka, India; Molecular Enzymology Laboratory, Department of Biosciences and Bioengineering, Indian Institute of Technology Bombay, Powai, Mumbai 400076, Maharashtra, India; Symbiosis School of Biological Sciences, Symbiosis International (Deemed University), Lavale, Pune 412115, Maharashtra, India

**Keywords:** *Aspergillus niger*, *Candida parapsilosis*, *Saccharomyces cerevisiae*, ureohydrolase, 3-guanidinopropionase, 4-guanidinobutyrase, substrate specificity

## Abstract

Enzymes of the ureohydrolase superfamily are specific in recognizing their substrates. While looking to broaden the substrate specificity of 4-guanidinobutyrase (GBase), we isolated a yeast, typed as *Candida parapsilosis* (NCIM 3689), that efficiently utilized both 4-guanidinobutyrate (GB) and 3-guanidinopropionate (GP) as a sole source of nitrogen. A putative GBase sequence was identified from its genome upon pBLAST query using the GBase sequence from *Aspergillus niger* (AnGBase). The *C. parapsilosis* GBase (CpGBase) ORF was PCR amplified, cloned, and sequenced. Further, the functional CpGBase protein expressed in *Saccharomyces cerevisiae* functioned as GBase and 3-guanidinopropionase (GPase). *S. cerevisiae* cannot grow on GB or GP. However, the transformants expressing CpGBase acquired the ability to utilize and grow on both GB and GP. The expressed CpGBase protein was enriched and analyzed for substrate saturation and product inhibition by γ-aminobutyric acid and β-alanine. In contrast to the well-characterized AnGBase, CpGBase from *C. parapsilosis* is a novel ureohydrolase and showed hyperbolic saturation for GB and GP with comparable efficiency (V_max_/K_M_ values of 3.4 and 2.0, respectively). With the paucity of structural information and limited active site data available on ureohydrolases, CpGBase offers an excellent paradigm to explore this class of enzymes.

## Teaser

The broad substrate specificity of 4-guanidinobutyrase enables *Candida parapsilosis* to efficiently utilize both 4-guanidinobutyrate and 3-guanidinopropionate as a sole nitrogen source.

## Introduction

Ureohydrolase superfamily (E.C. 3.5.3.x) consists of hydrolytic enzymes like arginase (E.C. 3.5.3.1), agmatinase, formiminoglutamase, proclavaminate amino hydrolase (PAH), 4-guanidinobutyrase (GBase; E.C. 3.5.3.7), and 3-guanidinopropionase (GPase; E.C. 3.5.3.17). These enzymes cleave off urea from their respective guanidinium group-containing substrates (with the exception of PAH) and they are very specific in recognizing their substrate side chains (Kumar et al. 2015). GBase catalyses the hydrolysis of 4-guanidinobutyrate (GB) and produces γ-aminobutyric acid (GABA) and urea. The GABA enters the TCA cycle by its conversion into succinate while the urea formed hydrolyzed to NH_3_ for use as the nitrogen source. Whereas, GPase catalyzes the hydrolysis of 3-guanidinopropionic acid (GP) to urea and β-alanine. The β-alanine is then metabolized via malonic semialdehyde and acetyl CoA to enter the TCA cycle; however, this pathway is not operational in all organisms. GBase from a few organisms is a broadly specific enzyme and can also act on GP. The metabolism of GP assumes significance as it is used as a dietary supplement in sports medicine (Oudman et al. 2013) and is being investigated as a potential antihypertensive drug (Karamat et al. 2015). However, there is only one report so far on a specific GPase in *P. aeruginosa* PAO1 (Lee et al. 2011).

In the ascomycetes fungus *Aspergillus niger*, two ureohydrolases namely, arginase and GBase were functionally annotated and fungal growth on different guanidium compounds was also characterized (Dave et al. 2012, Kumar et al. 2015). Interestingly, GB is efficiently utilized by *A. niger*, but GP is not. The *A. niger* GBase acts 25 times more efficiently on GB than on GP, and the *∆gbu* (GBase knockout) strain was unable to grow on GP (Saragadam et al. 2019). This suggested the absence of a specific GPase in this fungus. But *A. niger* can effectively utilize both urea and β-alanine, the two products of GP hydrolysis. And the GBase overexpressing *A. niger* strain shows better growth on GP. These observations collectively point to the poor GPase activity of *A. niger* GBase (AnGBase).

Our molecular understanding of ureohydrolase substrate specificity is still evolving (Dutta et al. 2019, Sekula 2020, Maturana et al. 2021, Funck et al. 2022). Not many GBase structures are available (Nakada and Itoh 2002, 2005, Lee et al. 2011), and hence rational substrate specificity engineering through site-directed mutagenesis is difficult. At first, we attempted to improve AnGBase specificity towards GP through EMS mutagenesis and screening of *A. niger* conidia, albeit with little success (Saragadam et al. 2019). A directed evolution approach (using error-prone PCR) was also undertaken; for this, *in vitro*, mutated AnGBase ORFs were transformed into *Saccharomyces cerevisiae* 12T7cI (*Δcar1::kanMX4, ura3*). The inability of *S. cerevisiae* to utilize GB, GP, and arginine (the *Δcar1* background) served well to set up a GPase screen. While screening for such transformants (around 1.0 lakh colonies; T. Saragadam, personal communication), a yeast contaminant that grew efficiently on GP was isolated. This new yeast isolate, a *Candida parapsilosis* strain, was evaluated for its GBase and GPase profiles. The characterization of a novel broad specificity GBase/GPase from this yeast is presented here.

## Material and methods

### Organisms and growth conditions

*Aspergillus niger* NCIM 565 (and its transformants) was maintained on potato dextrose agar slants. The yeast strains were maintained on yeast nitrogen base (YNB) agar supplemented with 5 mM ammonium sulfate (AS) and 2% glucose. *Saccharomyces cerevisiae* 12T7cI (*Δcar1::kanMX4, ura3*), an arginase-negative yeast mutant, was used to screen error-prone PCR products of *A. niger* GBase cDNA (Saragadam et al. 2019). Synthetic minimal medium [glucose 2.0 g, yeast nitrogen base (YNB and AS in 1:3 ratio) 0.66 g, distilled water 100 ml, agar 2.0 g] was used for this screening and the transformants were later replica plated on synthetic minimal medium supplemented with either 5 mM GB or GP instead of AS. The same medium without AS was used to grow *S. cerevisiae* 12T7cI transformants and the *C. parapsilosis* strain by supplementing an equimolar nitrogen source, namely, AS (7.5 mM), GB (5 mM), GP (5 mM), AR (3.75 mM), GABA (15 mM), β-alanine (15 mM), or urea (7.5 mM). Similar media without agar were prepared for liquid growth studies.

### Molecular techniques to manipulate DNA

QIAGEN DNeasy Plant Mini kit was used to prepare genomic DNA from yeast and *A. niger* for genomic PCR. Restriction digestions and ligations were performed essentially according to standard procedures (Sambrook and Russell 2001). The restriction enzymes and T4 DNA ligase were from New England Biolabs or MBI Fermentas. *Escherichia coli* strain XL1 Blue was used in all the transformation experiments for cloning purposes. Competent *E. coli* XL1 Blue cells were prepared by PEG method (Nishimura et al. 1990) and stored in glycerol/PEG at –80°C. Competent *E. coli* BL21 (DE3) and *E. coli* Rosetta gamiB (DE3) pLysS cells were prepared by CaCl_2_ method (Sambrook and Russell 2001) and stored at −80°C until further use.

When transformed with the desired plasmid, *E. coli* XL1-Blue, *E. coli* BL21 (DE3), and *E. coli* Rosetta gamiB were grown and maintained on Luria Bertani broth (containing 100 µg/ml ampicillin). Yeast transformation was performed by lithium acetate method (Ito et al. 1983). For this, the *S. cerevisiae* 12T7cI strain was inoculated into 10 mL YPD (Yeast extract 5 g/l, bactopeptone 10 g/l, and dextrose 20 g/l) medium. The plasmids bearing GBase expression constructs were linearized with *Sca*I and used to transform *A. niger* NCIM 565 protoplasts (Kumar et al. 2015). The transformants were single-spored and characterized for GBase expression.

### Identification of the yeast isolate as *C. parapsilosis*

The yeast isolate (eventually identified as *C. parapsilosis*) was grown in synthetic minimal medium supplemented with 5 mM AS, for genomic DNA isolation. The gDNA was isolated from a 24-h grown culture and was subjected to PCR amplification using ITS1 (5′-tccgtaggtgaacctgcgg-3′) and ITS4 (5′-tcctccgcttattgatatgc-3′) primers. The amplified PCR product was purified and sequenced using ITS1. The evolutionary history was inferred using the Maximum Parsimony (MP) method. The most parsimonious tree (length = 931) was generated with the consistency index of 0.631325, the retention index of 0.763524, the composite index of 0.512570 for all sites and the parsimony-informative sites (0.482032). The MP tree was obtained using the Subtree-Pruning-Regrafting algorithm with search level 1, in which the initial trees were obtained by the random addition of sequences (10 replicates). The analysis involved 24 nucleotide sequences. Codon positions included were 1st + 2nd + 3rd + Noncoding. There was a total of 586 positions in the final dataset. Evolutionary analyses were conducted in MEGA6. The yeast isolate was thereby identified as *C. parapsilosis*.

### Identification and cloning of putative GBase ORF from *C. parapsilosis*

The GBase and/or GPase loci are not functionally annotated in the *C. parapsilosis* genome. The *A. niger* GBase (AnGBase) protein sequence (AHL44994) served to search for putative GBase and/or GPase sequences in the *Candida* Genome Database (CGD). Based on the identity scores, forward (CparGPF; 5′-ccttgtccatatgaagttgcttccacttttaagc-3′) and reverse (CparGPR; 5′-gacctcgagtcagttggcacccttgtaaacttg-3′) primers were designed to PCR amplify the putative GBase ORF from *C. parapsilosis* gDNA. The *Nde*I and *Xho*I fragment of this amplicon was cloned in the pET.Nat.Arg vector by replacing *A. niger* arginase (Figure S1, Supporting Information). The resultant plasmid (pET23a-CparGB) was transformed in *E. coli* XL1 blue; the ampicillin-resistant transformants were screened and characterized by colony PCR. The plasmid isolated from these transformants were subjected to restriction digestion and sequencing. The cloned putative *C. parapsilosis* GBase (CparGBase) ORF (CPAR2_602270) was of 1104 bp (coding for a 367-residue polypeptide) with no introns predicted.

### Heterologous expression of *C. parapsilosis* GBase (CpGBase)

The pET23a-CparGB plasmid bearing CparGBase ORF (CPAR2_602270 sequence) was separately transformed in *E. coli* BL21 (DE3) and *E. coli* Rosetta gamiB (DE3) pLysS. The growth and induction were carried out according to standard procedures (Sambrook and Russell 2001). Ureohydrolases, by and large, are metalloenzymes with a bimetallic center at their active site. They contain Mn[II], Co[II], Ni[II], or Zn[II] at the active site (Funck et al. 2022). The use of His-tag for the expression and purification of such enzymes would complicate the interpretation of activity data. Hence the use of His-tag was avoided.

Attempts to express CpGBase in *S. cerevisiae*, a yeast closely related to *C. parapsilosis*, were made. The CpGBase ORF sequence was taken out from pET23a-CparGB (as *Nde*I-*Xho*I fragment) moved into a pBS vector (pBS-CparGB) and subsequently cloned as *Eco*RI-*Xho*I fragment in p426GPD (a plasmid used for constitutive protein expression in *S. cerevisiae*; Figure S1, Supporting Information). The resultant p426GPD-CparGB plasmid was transformed into *S. cerevisiae* 12T7cI strain (*Δcar1::kanMX4, ura3*); the p426GPD-GB (for expressing *A. niger* GBase) was also transformed in parallel, for comparison. The AnGBase cDNA was moved as an *Eco*RI-*Xho*I fragment into p426GPD to obtain p426GPD-GB (Saragadam et al. 2019). Yeast transformed with empty p426GPD vector served as control. The transformants were selected based on uracil auxotrophy and were maintained on synthetic minimal media supplemented with 5 mM of AS or GB.

Growth profiles of *C. parapsilosis* and *S. cerevisiae* transformants (Sc-Cp, expressing CpGBase or Sc-An, expressing AnGBase) on synthetic minimal medium appropriately supplemented with different nitrogen sources (5 mM each of AS, AR, GB, GP, GABA, urea, or β-alanine) were compared both by spot assay on agar media as well as by growth curve in liquid broth.

The CpGBase ORF was cloned in a pCB-vector for its constitutive expression in *A. niger*. The *Nde*I-*Not*I fragment bearing this ORF was cloned in front of the constitutive *A. niger citA* promoter (in pCBΔXCGB; Kumar et al. 2015) to obtain pCB-PcitA Cpar. This plasmid was linearized with *Sca*I and used to transform *A. niger* protoplasts. The transformants were single spored and characterized (by genomic PCR), for the integration of the plasmid. The transformants obtained along with the parent host strain (*A. niger* NCIM 565) were spot inoculated (10^3^ spores) on media containing different nitrogen sources (at 5 mM; ammonium nitrate, GB and GP) for growth comparison.

### Assay of different ureohydrolases

The arginase, GBase, and GPase activity in the cell-free extract was assayed using modified Archibald’s method of urea detection as described earlier (Saragadam et al. 2019). One unit of arginase, GBase, or GPase activity is defined as the amount of enzyme required to produce 1 µmol of urea per minute under the standard assay conditions. Specific activity is defined as units of enzymes per mg of protein.

The substrate saturations (with GB and GP) with enriched *C. parapsilosis* GBase (CpGBase) were performed using the 4-(dimethylamino)benzaldehyde (DMAB) method optimized for GBase and GPase activity (Supplementary Information S1; Figures S3–S7, Supporting Information). For the assay, the DMAB reagent was prepared by dissolving DMAB (4%, w/v) in absolute ethanol and sulfuric acid (4%, v/v) and stored at 4°C. The assay reaction includes phosphate buffer at 20 mM and GB (1–20 mM), and GP (1–40 mM). The reaction was initiated by adding the respective substrate and allowing the reaction to proceed for 20 min at 37°C. The reaction was stopped by adding 750 µl of DMAB reagent (freshly diluted with distilled water in a 1:2 ratio). These tubes were incubated at room temperature for 10 min, and absorbance at 420 nm was recorded.

### Enrichment of CpGBase

To determine the kinetic parameters of CpGBase, it was enriched from the cell-free extract of Sc-Cp strain (*S. cerevisiae* expressing CpGBase), using DEAE Sepharose and Superdex 200 columns sequentially. The DEAE Sepharose column (manually packed, 20 ml bed volume) was pre-equilibrated with phosphate buffer. The desalted protein sample was loaded onto this and binding was done at a flow rate of about 0.3 ml/min. After washing with 6 column volumes of buffer (flow rate of 2 ml/min) the column was eluted at the same flow rate using a 0 to 1 M KCl linear gradient. Fractions (2 ml each) were collected and analyzed for CpGBase activity. The peak fractions from the DEAE step were chosen (based on activity and native PAGE analysis), pooled and concentrated by subjecting to 0%–80% AS saturation. The protein pellet was dissolved in about 700 µl of buffer and proteins resolved on by gel filtration (Superdex 200, HiLoadTM 16/60 prep grade prepacked column, GE Healthcare). A 0.5-ml injection loop was used; the column was run at a flow rate of 1.0 ml/min at 25ºC. The fractions (1.0 ml, stored immediately at 4ºC) were assayed for CpGBase activity and analyzed on native PAGE.

### Electrophoretic procedures

Protein electrophoresis was performed in a Hoefer SE250 system according to the manufacturer’s instructions. Both the native PAGE (Davis 1964) and the SDS-PAGE (Laemmli 1970) were performed with minor modifications (Saragadam et al. 2019).

DNA electrophoresis was performed in the horizontal gel electrophoresis system (Bangalore Genei, Bangalore, India) following the manufacturer’s guidelines. The DNA was separated in 1% agarose gel with 0.5 µg/ml ethidium bromide prepared in 1X TBE buffer. The gel picture was recorded under UV using the Geliance 1000 Imaging system (Perkin Elmer, Waltham, MA, USA).

### Kinetic characterization of CpGBase

The peak fractions, chosen based on the activity and gel profile were used as the enriched CpGBase for kinetic studies. The enriched fractions were checked for saturation with GB (0.1–20 mM) and GP (2–40 mM) by DMAB assay. The data was plotted, and respective kinetic parameters were determined. The enriched CpGBase from Sc-Cp strain (grown on synthetic minimal media supplemented with 5 mM GB) was used for inhibition studies. The GBase and GPase activities were estimated by the modified Archibald’s method (Saragadam et al. 2019). GABA and β-alanine were used at varying concentrations from 0 to 200 mM.

### Protein estimation

Protein estimations were performed according to Bradford (1976), with bovine serum albumin as a reference.

### Homology modelling and bioinformatic analysis

The models to compare the overall structures and the active sites of AnGBase (AHL44994) and CpGBase (OK067409) were generated using the Alpha-Fold. These models were compared for their loop regions at the active site with the *Pseudomonas aeruginosa* PAO1 GBase (3NIO) and GPase (3NIP) structures.

## Results and discussion

### Identification of the yeast isolate that grows well on GP

**Figure 1.**
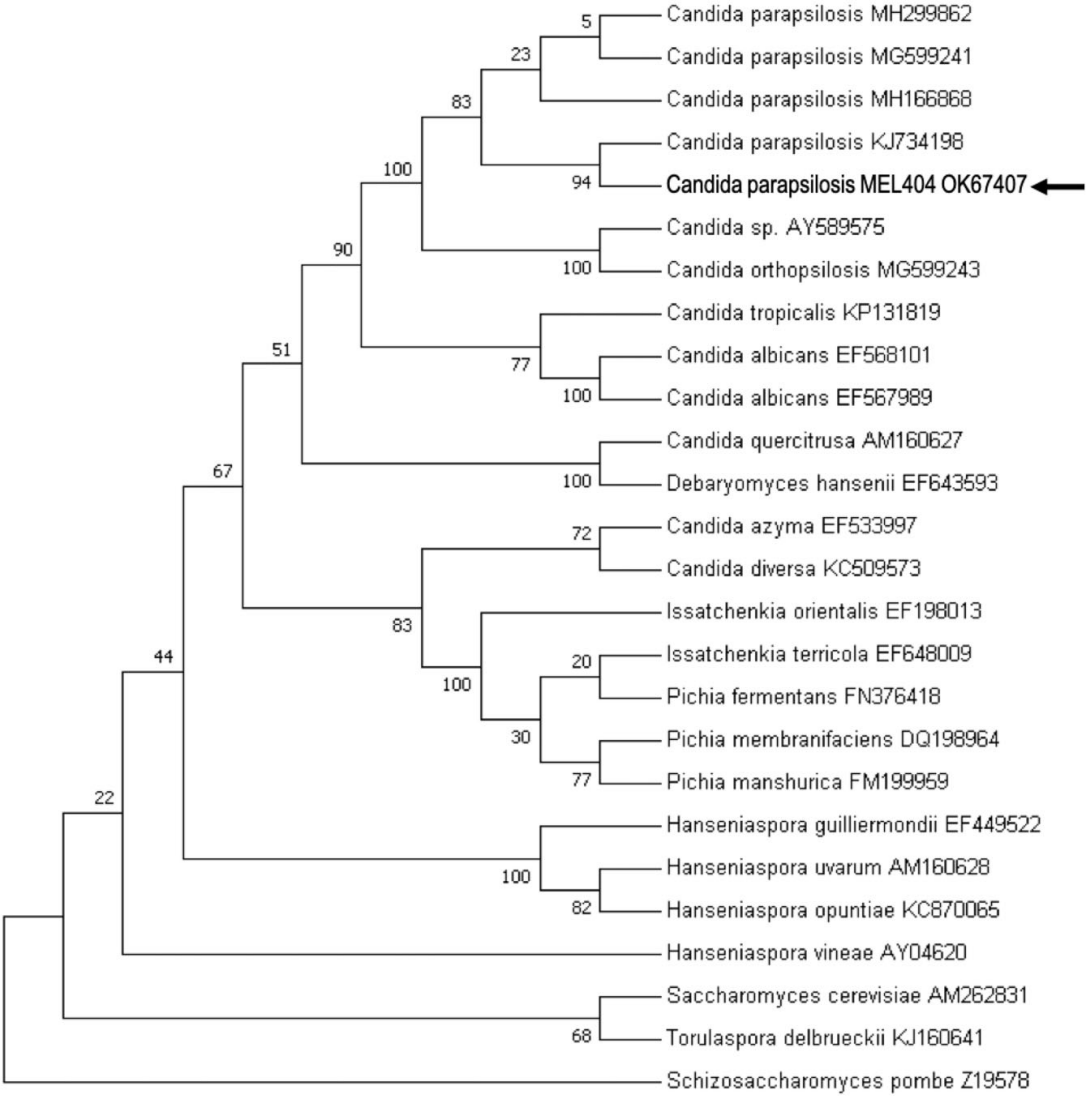
Phylogenetic analysis of the yeast isolate based on ITS1, 5.8S, 18S, and ITS4 rDNA sequences. The arrow shows the position of this yeast isolate (*C. parapsilosis* MEL404; deposited as NCIM 3689) in the phylogenetic tree. The isolate is most closely related to *C. parapsilosis* strains. The tree is rooted with *S. pombe* as an out-group and analysis details are given in the section ‘Material and methods’.

The yeast isolate that efficiently catabolized both GB and GP was characterized in detail. It was identified as *C. parapsilosis* using internal transcribed spacer (ITS) sequencing. That sequence was deposited in NCBI GenBank under the accession number OK067409. The phylogenetic relationship of the isolated *C. parapsilosis* strain with related yeast strains is shown in Fig. 1. The *C. parapsilosis* isolate, designated as the MEL404 strain, was deposited at the National Collection of Industrial Microorganisms (NCIM), Pune, India, under the accession number NCIM 3689.

### Profiling different ureohydrolase activities of *C. parapsilosis* NCIM 3689

The yeast isolate, *C. parapsilosis* NCIM 3689, was grown on AS, GB, GP, and AR and the respective cell-free extracts were assayed for arginase, GBase, and GPase activities. None of the three activities were detected when this yeast was grown on AS. However, all three activities were detected in GB and GP grown yeast cells. The GBase and GPase activities were strictly induced in presence of GB or GP. Arginase activity was induced to some extent when GB or GP was the sole nitrogen source. The specific activity ratios of GPase:GBase, were similar in *C. parapsilosis* grown on GB and GP; suggesting that a single enzyme functions equally well, both as GBase and GPase in this yeast (Table 1). With the exception of *P. aeruginosa* PAO1 GPase, the GPase activity reported in all other cases is due to a broadly specific GBase (Yorifuji et al. 1982, Lee et al. 2011). Most broad specificity GBases display poor GPase activity (Saragadam et al. 2019). The *C. parapsilosis* enzyme appears to be an exception and therefore it was further characterized.

**Table 1.** Ureohydrolase activities of *C. parapsilosis* grown on different nitrogen sources.

	Specific activity (U/mg)			
Grown on	Arginase	GBase	GPase	GPase/GBase
AS	ND	ND	ND	
GB	3.03 ± 0.36	5.95 ± 0.64	6.15 ± 0.7	1.03
GP	2.80 ± 0.4	5.04 ± 0.52	5.25 ± 0.58	1.04
AR	11.93 ± 1.2	ND	ND	

AS, ammonium sulfate; GB, 4-guanidinobutyrate; GP, 3-guanidinopropionic acid; AR, arginine; GBase, 4-guanidinobutyrase; and GPase, 3-guanidinopropionase

The structural genes responsible for the GBase and/or GPase activities are not functionally annotated in *C. parapsilosis*. The *A. niger* GBase (AnGBase) protein sequence (Kumar et al. 2015) was used to search for putative GBase or GPase sequences in the CGD. The BLAST results showed three putative ORFs (Table 2). CPAR2_602270 ORF (with a maximum identity of 50.4%) was chosen for PCR amplification.

**Table 2.** Protein BLAST of *C. parapsilosis* genome queried with AnGBase sequence.

Hit ID	Hit organism	Length (aa)	% Identity
CPAR2_602270	*C. parapsilosis* CDC317	367	50.4
CPAR2_808380	*C. parapsilosis* CDC317	438	41.7
CPAR2_100820	*C. parapsilosis* CDC317	316	28.5

### Heterologous expression of *C. parapsilosis* GBase in *E. coli*

The pBLAST with AnGBase sequence as the query yielded three hits in the *C. parapsilosis* genome (from CGD) (Table 2). The putative *C. parapsilosis* GBase (CpGBase) ORF (CPAR2_602270) with the highest identity was 1104 bp long and was without introns. Based on this sequence, primers (CparGPF and CparGPR) were designed with *Nde*I and *Xho*I restriction sites for cloning this ORF. The putative CpGBase ORF was amplified using CparGPF and CparGPR primers and cloned in pET23aNat.Arg plasmid by replacing the arginase sequence between the *Nde*I and *Xho*I sites (Figure S1, Supporting Information). The resultant plasmid (pET23a-CparGB) was transformed in *E. coli* XL1 blue. The ampicillin-resistant transformants were screened and characterized by colony PCR and restriction digestion. The positive transformants were further confirmed by sequencing the relevant plasmids. The pET23a-CparGB plasmid bearing CPAR2_602 270 sequence was transformed in *E. coli* BL21 (DE3).

**Figure 2.**
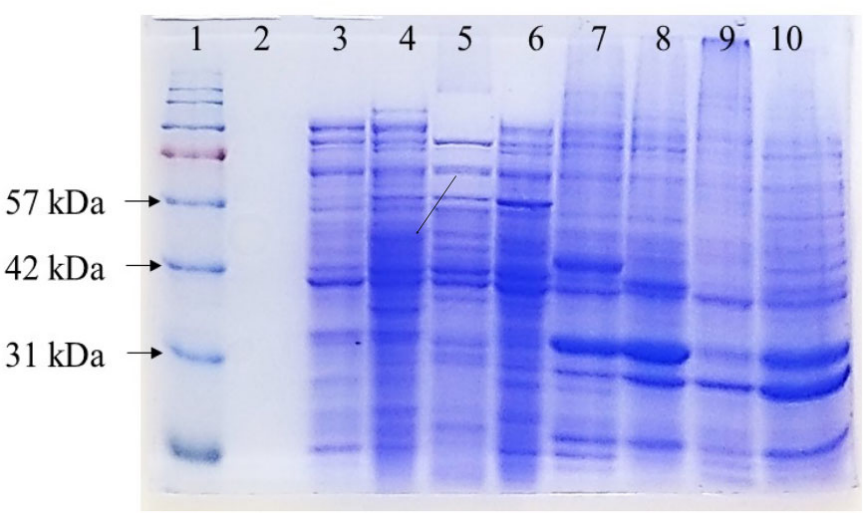
Heterologous expression analysis of CpGBase and AnGBase by SDS-PAGE. Various *E. coli* protein fractions before and after IPTG induction are shown. Lane 1: molecular weight markers, 3: AnGBase-uninduced supernatant, 4: AnGBase-induced supernatant, 5: CpGBase-uninduced supernatant, 6: CpGBase-induced supernatant, 7: AnGBase-uninduced pellet, 8: AnGBase-induced pellet, 9: CpGBase-uninduced pellet, and 10: CpGBase-induced pellet. Lane 2 is blank; the arrow indicates the band corresponding to AnGBase.

The expression of a functional CpGBase in *E. coli* BL21 (DE3) could not be detected by the standard GBase assay under various IPTG induction and growth conditions. The SDS-PAGE of the cell-free extracts also did not show the protein of expected size (∼40 kDa; a 367 aa polypeptide). Under similar conditions, however, the AnGBase protein (∼50.4 kDa; a 422 aa polypeptide) was expressed efficiently (Fig. 2) and was also functional (Saragadam et al. 2019). Attempts were also made to express CpGBase in *E. coli* rossetta gamiB cells so as to rule out the possibility of the expressed protein toxicity. However, no GBase activity or protein could be detected.

### CpGBase expression in *A. niger*

The earlier growth showed that *A. niger* could efficiently utilize GB and β-alanine as the sole nitrogen sources, whereas GP was poorly utilized. Also, AnGBase showed poor substrate specificity towards GP (Saragadam et al. 2019). However, the overexpression of AnGBase in *A. niger* showed improved growth on GP, suggesting that the growth on GP is limited by poor GPase activity of AnGBase. The heterologous expression of a functional CpGBase was anticipated to facilitate the growth of *A. niger* on GP. Accordingly, the CpGBase ORF was cloned in a pCB-vector under the constitutive *PcitA* to obtain pCB-*PcitA*Cpar (Figure S2, Supporting Information). This plasmid was linearized with *Sca*I and used to transform *A. niger* protoplasts (Kumar et al. 2015). After five passages on the selection medium (YDA supplemented with phosphinothricin) 18 stable transformants were obtained and four of them ((T3, T8, T13, and T14) were selected for further characterization by genomic PCR and by growth on different nitrogen sources.

**Figure 3.**
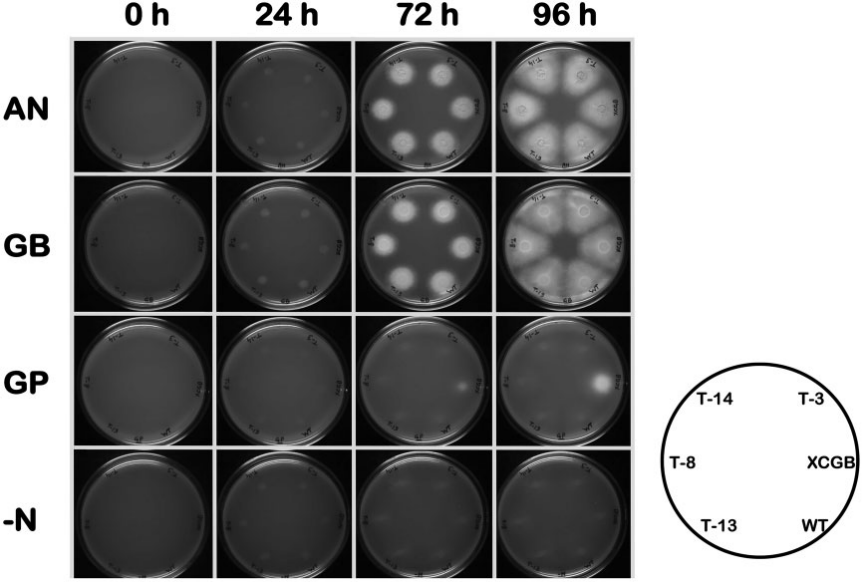
Growth of *A. niger* transformants on various nitrogen sources. The *A. niger* isolates (transformed with pCB-*PcitA*Cpar and anticipated to heterologously express CpGBase) numbered as T-3, T-8, T-14, and T-13, were spot inoculated (10^3^ spores each) on YNB agar alone or supplemented (individually at 5 mM) with ammonium nitrate, GB and GP. The *A. niger* strain (XCGB13) overexpressing AnGBase served as a control. The bottom right corner shows the key of the strains inoculated.

The *A. niger* CpGBase transformants, XCGB13 strain (overexpressing AnGBase) and the parent *A. niger* NCIM 565 were spot inoculated on minimal media supplemented with different N-sources (5 mM of ammonium nitrate, GB or GP). All CpGBase transformants showed comparable growth on ammonium nitrate and GB, similar to that of XCGB13 strain and the parent strain (Fig. 3). While the XCGB13 strain grew on GP plates (albeit slowly), none of the CpGBase transformants could do so. When the CpGBase transformants grown on minimal media (and their mycelial extracts were analyzed) none displayed GBase activity. The XCGB13 strain (as a positive control) did show GBase activity as expected (Kumar et al. 2015).

### CpGBase expression in *S. cerevisiae*

The attempts to express putative CpGBase ORF in *E. coli* and *A. niger* were not fruitful. On the contrary, despite being a longer polypeptide (422 residues versus the 367 residues CpGBase), the AnGBase could be successfully expressed in both these organisms (Kumar et al. 2015, Saragadam et al. 2019). This could be due to—(a) the codon bias between *C. parapsilosis* and these two expression hosts and/or (b) the instability of the expressed CpGBase protein. The *C. parapsilosis* GBase sequence, although belonging to the CTG clade (Papon et al. 2013), does not have a single CTG codon in the ORF. It is less likely, therefore, that codon bias (residue changes to Ser in place of Leu) may be involved, while protein stability could be the cause for CpGBase nonexpression in both *E. coli* and *A. niger* hosts. Further, an attempt was made to express CpGBase in *S. cerevisiae* (another yeast related to *C. parapsilosis* but that lacks endogenous GBase). The plasmid p426GPD, furnished with the constitutive gpd promoter, was used for this expression. The *S. cerevisiae* 12T7cI strain (*Δcar1::kanMX4, ura3*) is unable to utilize GB, GP and arginine and also provides for *ura* selection. The *Δcar1* background ensured that the major ureohydrolase (i.e. arginase) is absent to facilitate—(a) clean screening of transformants and (b) monitoring the substrate specificity of expressed GBase through plate growth assays. Towards this, the plasmid p426GPD-CparGB was then transformed in *S. cerevisiae* 12T7cI strain; in parallel, the p426GPD-GB (for expressing *A. niger* GBase) was also transformed for comparison (see the section ‘Methods’). Yeast transformed with an empty p426GPD vector served as control.

**Figure 4.**
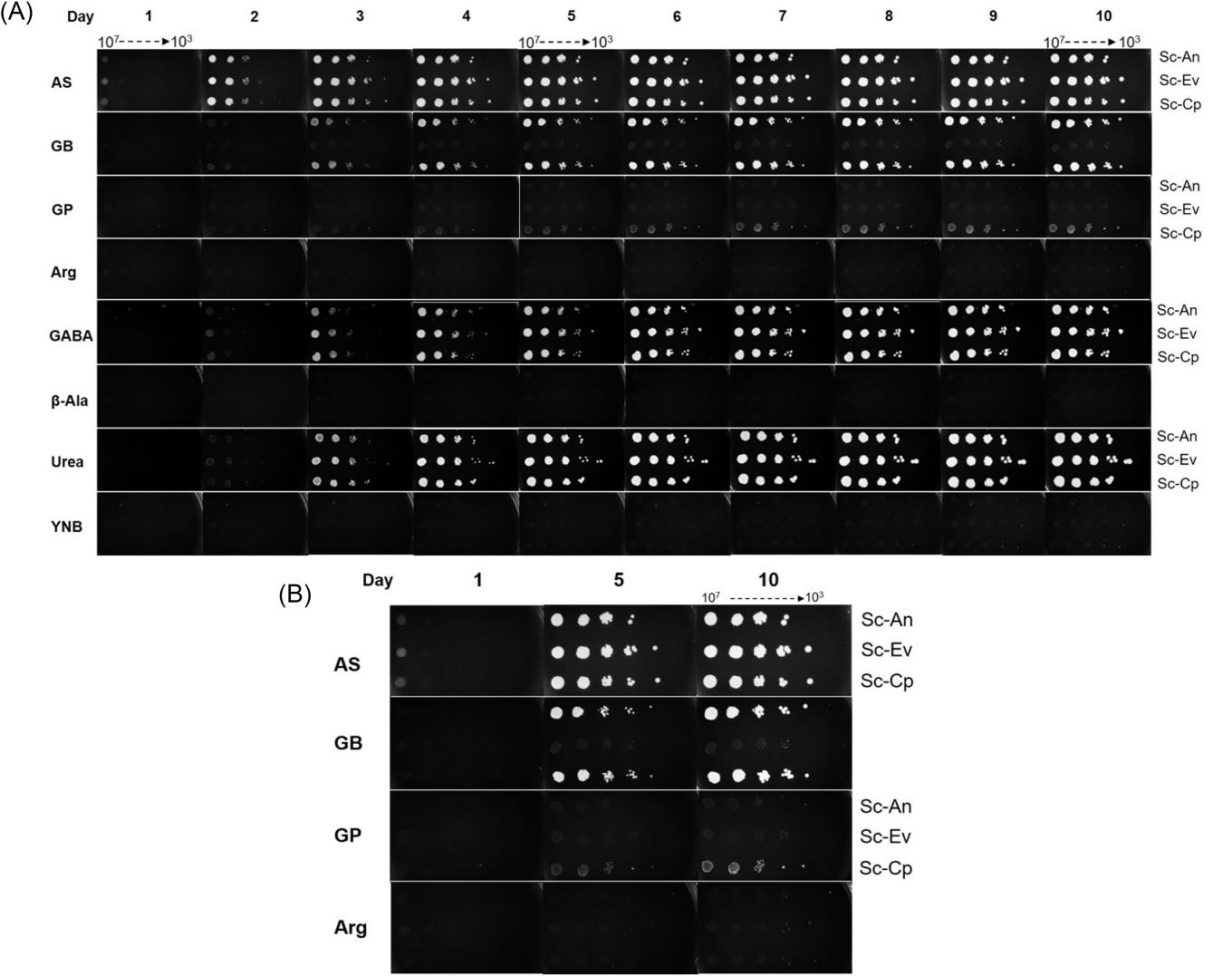
Growth of Sc-An and Sc-Cp transformants on different nitrogen sources. (A) *Saccharomyces cerevisiae* transformants expressing AnGBase (Sc-An) and CpGBase (Sc-Cp) were spot inoculated (serial dilutions of 10^7^–10^3^ cells/ml) on AS, GB, GP, AR, GABA, β-Ala, Urea, and YNB. All the nitrogen sources were present at 5 mM and plates were incubated at 30°C, and the images were captured every 24 h. Yeast transformed with an empty vector (Sc-Ev strain) served as a control. (B) Growth data from days 1, 5, and 10 are highlighted, with growth on AS and Arg as controls.

The *S. cerevisiae* transformants bearing p426GPD-CparGB (abbreviated as Sc-Cp strain, expressing CpGBase) showed both GBase and GPase activity and also acquired the ability to utilize GB or GP, indicating that CpGBase ORF was successfully expressed in *S. cerevisiae* and that the expressed protein was functional. Similarly, Sc-An (expressing AnGBase) and Sc-Ev (empty vector control) transformants were also tested. The Sc-An strain but not the Sc-Ev strain displayed GBase activity. The growth of the three *S. cerevisiae* transformants (Sc-Cp strain, Sc-An strain, and Sc-Ev strain) was compared by spot growth assays and through growth curves. *S. cerevisiae* naturally cannot utilize GB and GP as the sole nitrogen source. Inability of Sc-Ev strain to grow either on GB or GP plates is consistent with this observation. Yeast transformants expressing AnGBase (Sc-An strain) or CpGBase (Sc-Cp strain) were able to grown on and utilize GB efficiently. However, only the Sc-Cp strain could utilize GP as a nitrogen source, albeit poorly (Fig. 4). A relatively modest growth on GP could be due to poor transport/uptake of GP and the inability of *S. cerevisiae* (unlike *C. parapsilosis*) to utilize β-alanine as a nitrogen source. It is possible that other putative enzymes in *C. parapsilosis* could impart better growth for this yeast on GP. It is known from literature that *S. cerevisiae* cannot utilize β-alanine (one product of GPase reaction). Yet, *S. cerevisiae* growth on GP must exploit the other product urea as the available nitrogen source. Also, relative rates of urea utilization between the two yeasts could be different. Expressed CpGBase conferring growth advantage to *S. cerevisiae* on GP is however clearly established by this study.

**Figure 5.**
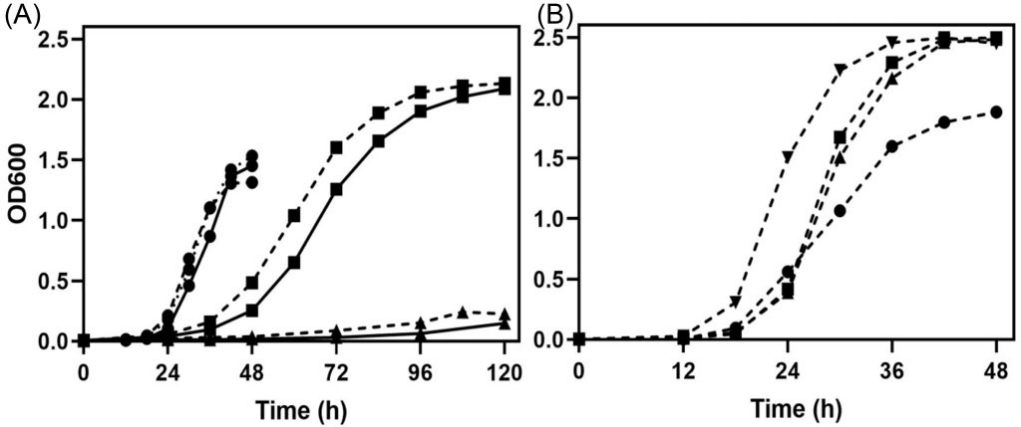
Growth of *C. parapsilosis* and *S. cerevisiae* transformants in liquid culture. (A) The *S. cerevisiae* transformants Sc-Ev (⋯), Sc-An (—), and Sc-Cp (**⁃⁃⁃**) yeast cultures were grown (with 0.5 × 10^7^ cells/ml as the inoculum) on different nitrogen sources (all at 5 mM), namely, AS(●), GB(■), GP(▲), or AR(▼) in liquid synthetic minimal medium. AR(▼) is not plotted in panel (A) to avoid crowding, as there was no growth on this N source (the parent strain is *∆car1*). (B) Growth of *C. parapsilosis* NCIM 3689 (isolated strain) on different nitrogen sources in liquid culture (conditions and symbols are same as in A). Note: None of these organisms could grow on YNB alone.

The growth profile of *S. cerevisiae* transformants (Sc-Ev, Sc-An, and Sc-Cp) in liquid culture is shown in Fig. 5(A). All three strains utilized AS efficiently, and the growth reached the stationary phase within 48 h. The growth curve on AS for the isolated yeast (*C. parapsilosis* NCIM 3689) was also comparable (Fig. 5B). However, the growth of both the Sc-An and Sc-Cp strains was much slower on GB than on AS, with the Sc-Cp strain clearly performing better. Both grew very poorly on GP, while their relative growth was consistent with that observed on agar plates (Fig. 4B). The growth difference between Sc-An and Sc-Cp, on GB as a sole source of nitrogen, is more prominent in submerged culture than that observed on agar plates. In contrast to the two *S. cerevisiae* transformants, the isolated *C. parapsilosis* strain efficiently utilized both GB and GP and attained the stationary phase within 48 h. In fact, the growth of *C. parapsilosis* on all four nitrogen sources (including AS and AR) was comparable. Interestingly, the same CpGBase expressed in *S. cerevisiae*, while functional, is unable to provide growth advantage on GP to the host. This is not a reflection of differences in enzyme expression level (significant levels of CpGBase, both as GBase and GPase activities, could be detected in the cell-free extracts of *S. cerevisiae* transformant). Hence, the observed poor growth could be due to poor transport/uptake of GP and the inability of the *S. cerevisiae* host strain (unlike the parent *C. parapsilosis*) to utilize ꞵ-alanine as a nitrogen source. While the two organisms can effectively utilize GB, clearly, *C. parapsilosis* (NCIM 3689 isolate) is well endowed to assimilate GP also—a feature unlike *A. niger* (Saragadam et al. 2019). For these reasons, it was worth characterizing the properties of CpGBase in more detail.

### Characterization of *C. parapsilosis* GBase

**Figure 6.**
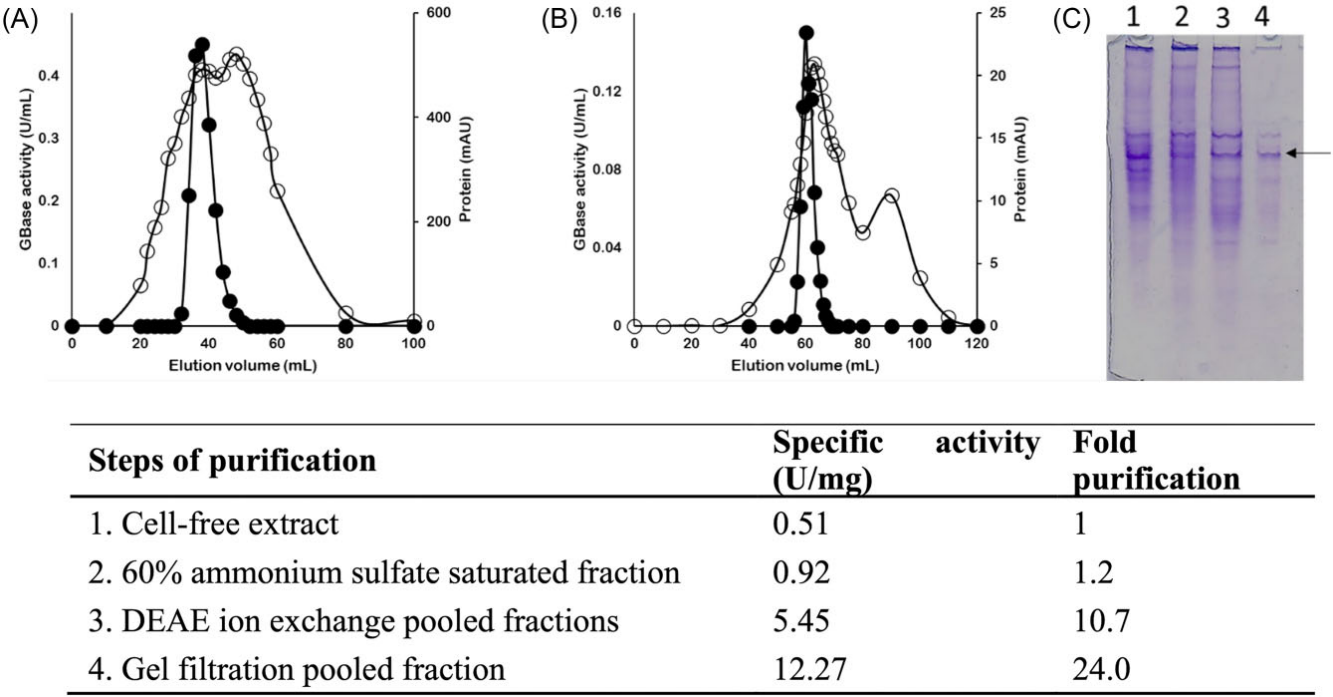
Purification of CpGBase expressed in *S. cerevisiae*. Elution profiles of GBase activity (●) and protein (○) from the DEAE Sepharose column (A) and Superdex 200 gel filtration column (B) are shown. The native PAGE of four different CpGBase fractions (1, 2, 3, and 4 in the table) collected at different stages of enrichment is also shown (C). The arrow in (C) points to the potential CpGBase protein band based on its specific activity increase after each step of purification (activity-guided purification), as shown in the table.

We noted earlier that the GPase activity of *C. parapsilosis* may be due to a single broadly specific GBase (Table 1). While *C. parapsilosis* could efficiently utilize GP through the activity of this enzyme, heterologous expression of CpGBase (and not AnGBase!) did manifest some growth advantage to *S. cerevisiae* transformants. Only a handful of GBases have been studied so far and AnGBase is one of them (Saragadam et al. 2019). It was, therefore, of interest to purify, kinetically characterize and compare the CpGBase with AnGBase. Despite some efforts, a functional CpGBase enzyme could not be expressed in *E. coli*. It was, however, possible to do so in the yeast background. Accordingly, CpGBase was expressed and enriched 24-fold from cell-free extracts of Sc-Cp strain (*S. cerevisiae* expressing CpGBase; see the section ‘Methods’), using DEAE Sepharose and Superdex 200 columns and the peak fractions were analysed on a native-PAGE gel (Fig. 6).

**Figure 7.**
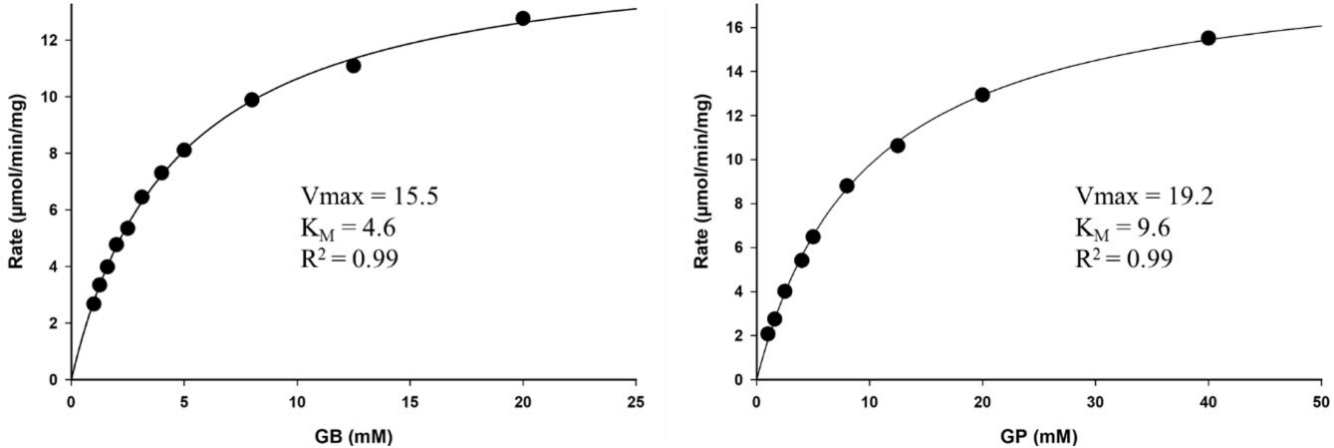
*Candida parapsilosis* GBase substrate saturation kinetics. The CpGBase enzyme assays for initial velocity analyses were performed in triplicate. The kinetic data is representative of three independent purifications.

The enriched fraction of CpGBase displayed both GBase and GPase activities. The enzyme was used for GB (0.1–20 mM) and GP (2–40 mM) saturations and using a DMAB assay. Both GB and GP saturations showed typical Michaelis–Menten curves (Fig. 7), and the V_max_/K_M_ values (3.4 and 2.0, respectively) were similar. The kinetic features of CpGBase were compared with those of the better-characterized AnGBase, and these are listed in Table 3. Clearly, AnGBase acts 30 times better on GB than GP, whereas CpGBase is just about twice as effective on GB than GP. Unlike AnGBase, CpGBase is thus a broad specificity enzyme and acts equally well on both GB and GP.

**Table 3.** A summary of kinetic parameters for AnGBase and CpGBase.

				K_I_	
Enzyme	Activity	K_M_ (mM)	Vmax/KM	GABA (mM)	β-alanine (mM)
AnGBase	GBase	2.7 ± 0.4	6.1	4.1 ± 1.1	48.7 ± 0.8
	GPase	53.7 ± 0.8	0.2	3.2 ± 0.8	33.9 ± 1.2
CpGBase	GBase	4.6 ± 0.5	3.4	3.1 ± 0.5	7.8 ± 1.2
	GPase	9.6 ± 0.4	2.0	3.4 ± 0.8	9.2 ± 1.4

**Figure 8.**
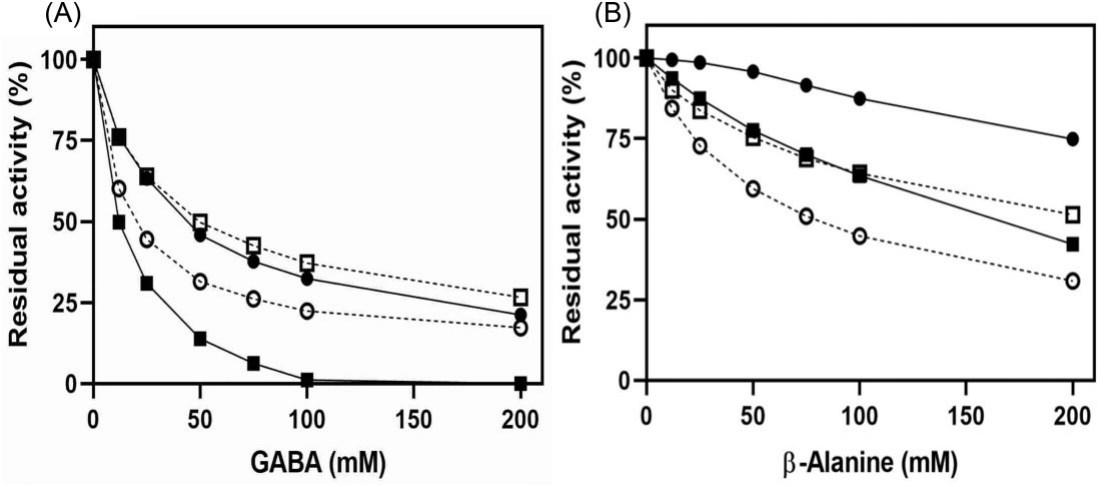
Inhibition of AnGBase and CpGBase activities by the reaction products. The GBase (●) and GPase (■) activity of AnGBase (—) and CpGBase (**⁃⁃⁃**) was performed in the presence of varying concentrations of GABA (A) and β-alanine (B). The activity without an inhibitor was considered 100% and plotted by calculating the respective residual activity (%) with increasing inhibitor concentration. Inhibition curves are representative of three separate experiments.

Product inhibition analysis can throw further light on the specificity aspects of CpGBase. Therefore, inhibition of the GBase and the GPase activities of CpGBase by their respective products (namely, GABA from GB hydrolysis and β-alanine from GP hydrolysis) was studied. Similar product inhibition data for AnGBase was used for comparison. GABA inhibited both the GBase and the GPase activity of CpGBase equally well. However, GABA was a strong inhibitor of the GPase activity of AnGBase than its GBase activity. Although β-alanine is a weak inhibitor when compared to GABA, it also showed a similar pattern of inhibition—being more effective on CpGBase (Fig. 8). Analysis of the inhibition data (Cheng and Prusoff 1973) was done to evaluate the respective K_I_ values for both GABA and β-alanine. A summary of inhibition results is given in Table 3. The inhibition patterns again points to the broader substrate specificity of CpGBase than AnGBase.

### Structural insights into *C. parapsilosis* GBase

**Figure 9.**
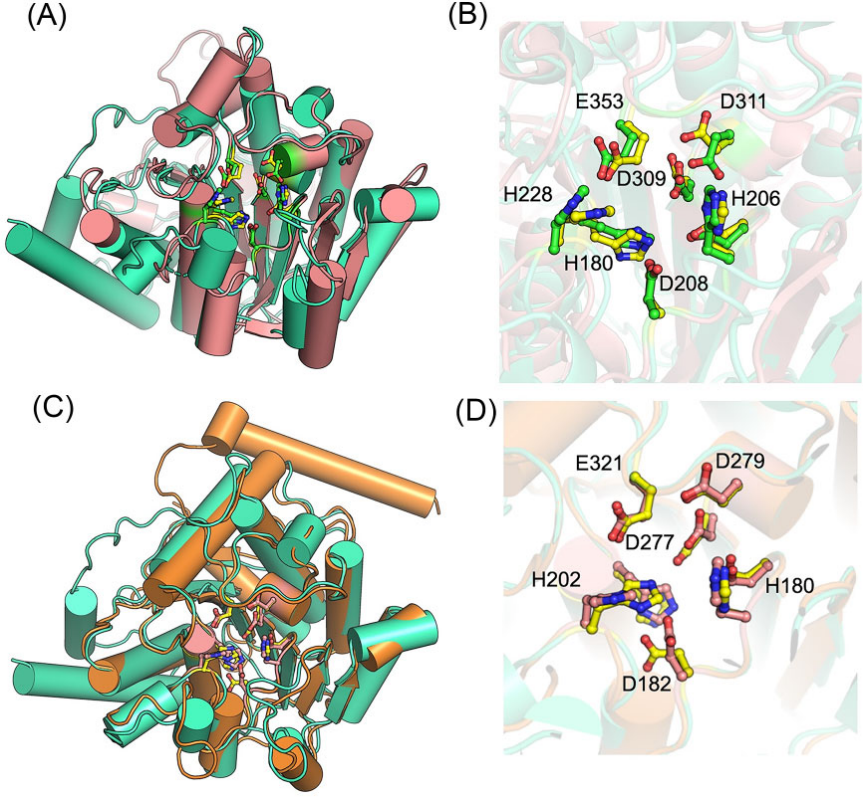
Structural models and active site residues of AnGBase and CpGBase. The *P. aeruginosa* PAO1 GBase (salmon) structure compared with AnGBase (cyan) model for the overall subunit structure (A) and the active site with AnGBase residues labelled (B). The CpGBase (orange) model compared with AnGBase subunit structure (C) and the corresponding active site (CpGbase residues labelled) (D).

Unlike AnGBase (a 422-residue polypeptide and highly specific towards GB), the CpGBase is a smaller protein (367 residues) and acts efficiently both on GB and GP. Identifying the residues critical for their substrate specificity is useful. It was, therefore, of interest to model and compare the two ureohydrolase structures. The structural data on ureohydrolases is largely limited to arginases and currently only one GBase structure is available (Lee et al. 2011). This GBase is reported to be a 319-residue polypeptide that functions as a homotetramer. Therefore, the structural models of both AnGBase and CpGBase were built using the Robetta platform (an online tool that is unbiased towards any single structure while building the models). The two proteins were first analyzed for their amino acid sequence similarity (Figure S8, Supporting Information). The AnGBase model obtained was comparable with its overall structural fold (α–β sandwich) and the active site geometry of the reported *P. aeruginosa* PAO1 GBase structure (Fig. 9A). The AnGBase structure was then used to compare with that of modeled CpGBase structure. AnGBase sequence (with 422 residues) is longer than that of CpGBase (with 367 residues). Besides the sequences contributing to the common structural fold (the α–β sandwich), additional sequence(s) in the AnGBase were found away from the active site (Fig. 9B).

**Figure 10.**
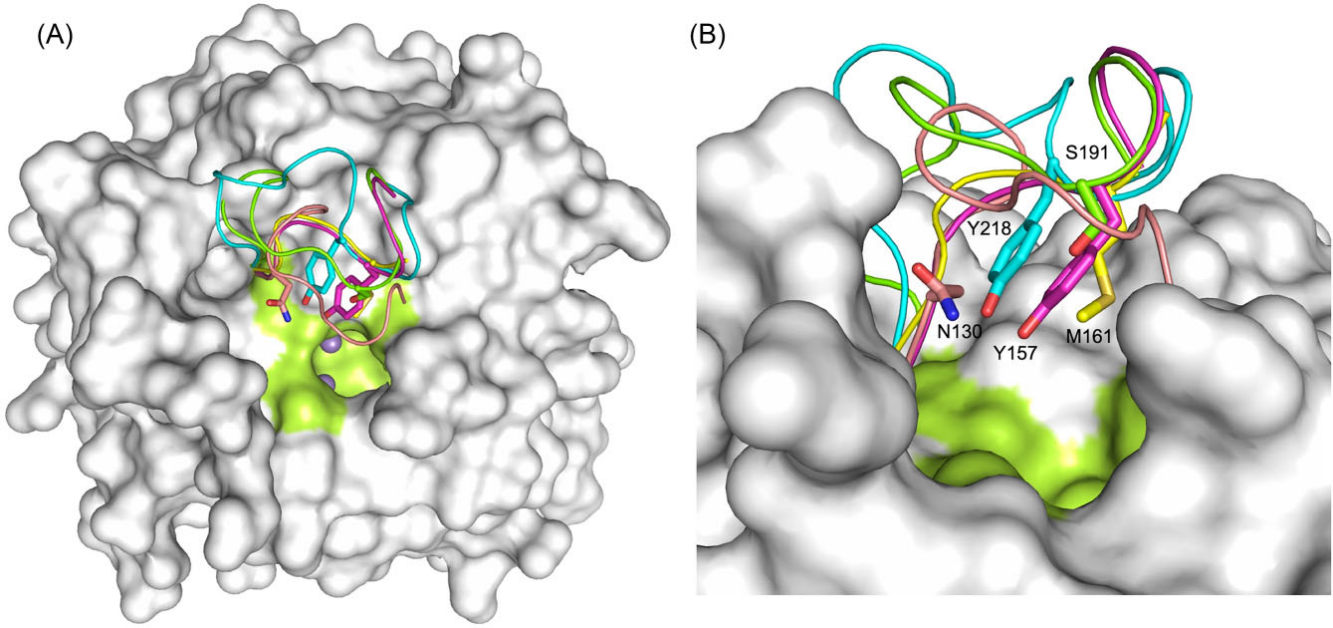
The active site loop regions of four different ureohydrolases. (A) The active site loop regions of CpGBase model (in green; top view) compared with those from human arginase-I (salmon), *P. aeruginosa* PAO1 GBase (yellow), *P. aeruginosa* PAO1 GPase (magenta) and AnGBase (cyan). (B) The same active site viewed at 90° angle with respective critical substrate recognition residues.

Like in the case of most known ureohydrolase structures, the active site in both AnGBase and CPBGase is largely built with flexible loops (Fig. 10). The differences in the length and residues in the loop regions covering the active site account for the substrate entry and specificity. The corresponding loop regions of human arginase-I, *P. aeruginosa* PAO1 GBase, and *P. aeruginosa* PAO1 GPase were compared with those predicted in the AnGBase and CpGBase models. The residues N130, M161, and Y157 in human arginase I, *P. aeruginosa* GBase, and GPase, respectively, were shown to be involved in substrate recognition (Alarcon et al 2006, Lee et al 2011). The corresponding loop residue was Y218 in AnGBase and S191 in CpGBase. The residue with a relatively small R group (S191 in CpGBase) could possibly accommodate both GB and GP in the active site and may account for the broader substrate specificity of CpGBase (Fig. 10). These possibilities could be tested by suitable site-directed mutagenesis studies of the two enzymes.

## Conclusions

The detailed kinetic characterization of CpGBase, showed its broader substrate-specificity when compared to AnGBase. CpGBase is a novel ureohydrolase, i.e. equally efficient with both GB and GP as substrates and is the first of its kind to be reported from fungi. A comparison of structural models derived from CpGBase and AnGBase sequences points to their common structural folds with active sites defined largely by loop regions. Hence, predicting and/or defining substrate specificity of ureohydrolases through docking is a daunting task. Along with *P. aeruginosa* GBase (319 amino acids) and AnGBase (422 amino acids), the CpGBase (367 amino acids) now provides an excellent paradigm to explore the structures and active sites of ureohydrolases in general and GBases in particular. Overall, the present work adds a new yeast enzyme to the list of ureohydrolase superfamily group; unlike other enzymes of the family, this enzyme has wide substrate specificity and different catalytic properties. Finally, the broad substrate specificity of CpGBase, in combination with GP as its substrate, has the potential to serve as a novel nutritional selection marker for fungal transformations.

## Supplementary Material

foae003_Supplemental_File
