# Supplementary material for: Characterization of a novel 4-guanidinobutyrase from Candida parapsilosis
Source: FEMS Yeast Res. 2024 Jan 18;24:foae003. doi: 10.1093/femsyr/foae003 (PMC10833137; doi:10.1093/femsyr/foae003)
Supplement: foae003_Supplemental_File [file foae003_supplemental_file.docx]

**Supplementary Information**

**Characterization of a novel 4-guanidinobutyrase from *Candida parapsilosis***

Santoshkumar R. Gaikwad^1^, Narayan S. Punekar^1,2^ and Ejaj K. Pathan^*1,3^

^1^Molecular Enzymology Laboratory, Department of Biosciences and Bioengineering, Indian Institute of Technology Bombay, Powai, Mumbai-400076

^2^Department of Biosciences and Bioengineering, Indian Institute of Technology Dharwad, Dharwad, Karnataka, India-580011

^3^Symbiosis School of Biological Sciences, Symbiosis International (Deemed University), Lavale, Pune, Maharashtra, India-412115

**Running title:** 4-Guanidinobutyrase from *Candida parapsilosis*

***Corresponding author:**

Dr. Ejaj K Pathan

Symbiosis School of Biological Sciences,

Symbiosis International (Deemed University),

Lavale, Pune, Maharashtra, India-412115

E-mail: [ek.pathan@ssbs.edu.in](mailto:ek.pathan@ssbs.edu.in); [ejaj2185@gmail.com](mailto:ejaj2185@gmail.com)

Phone: +91-20-28116365

**Supplementary Figure S1.** *C. parapsilosis* GBase amplification and cloning strategy. Putative *C. parapsilosis* GBase ORF amplified and cloned in pET23a vector by replacing Native arginase ORF. The *C. parapsilosis* GBase amplicon was moved as a *EcoR*I-*Xho*I fragment into the p426GPD vector resulting p426GPD-CparGBase for expression in yeast.


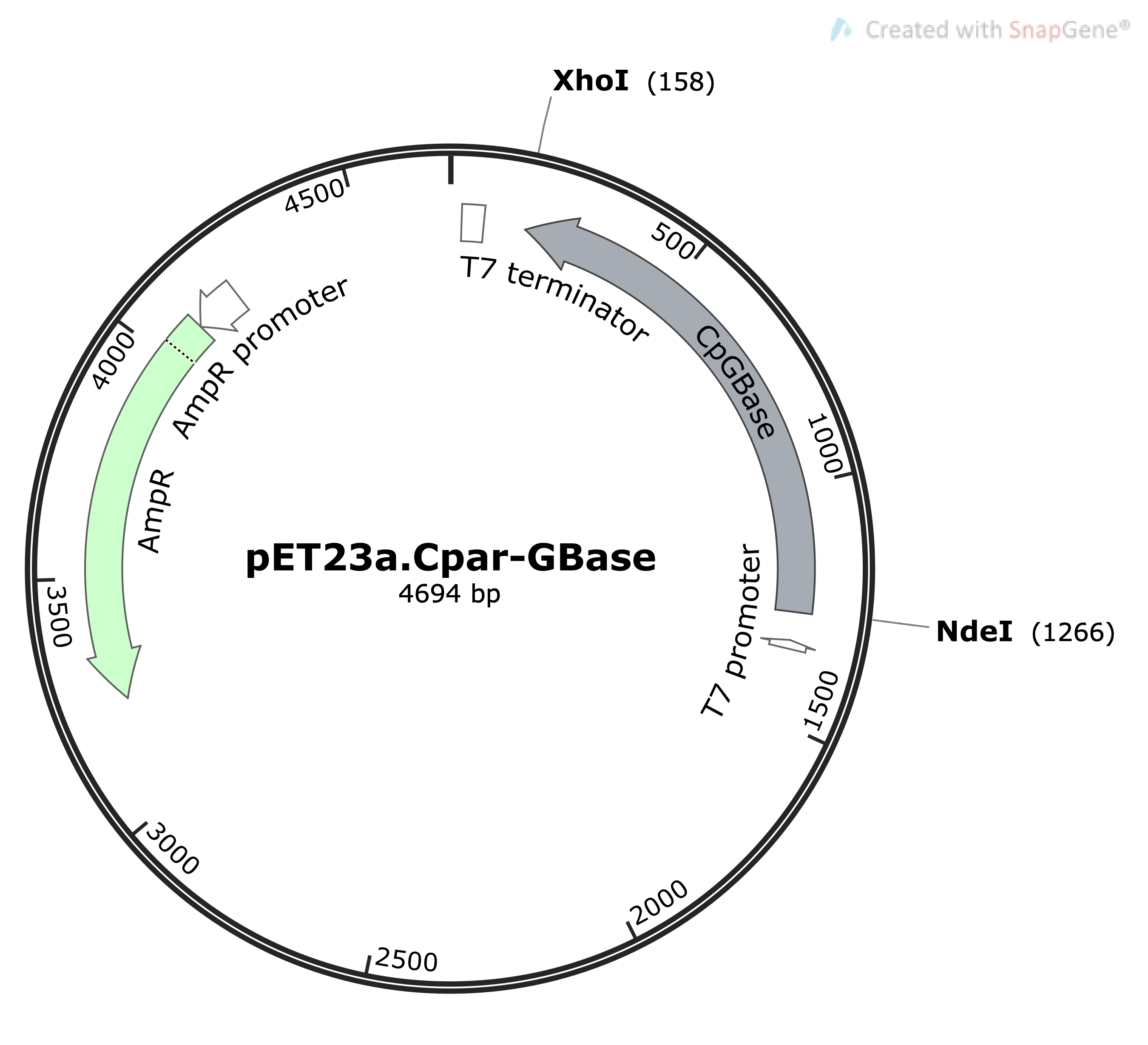

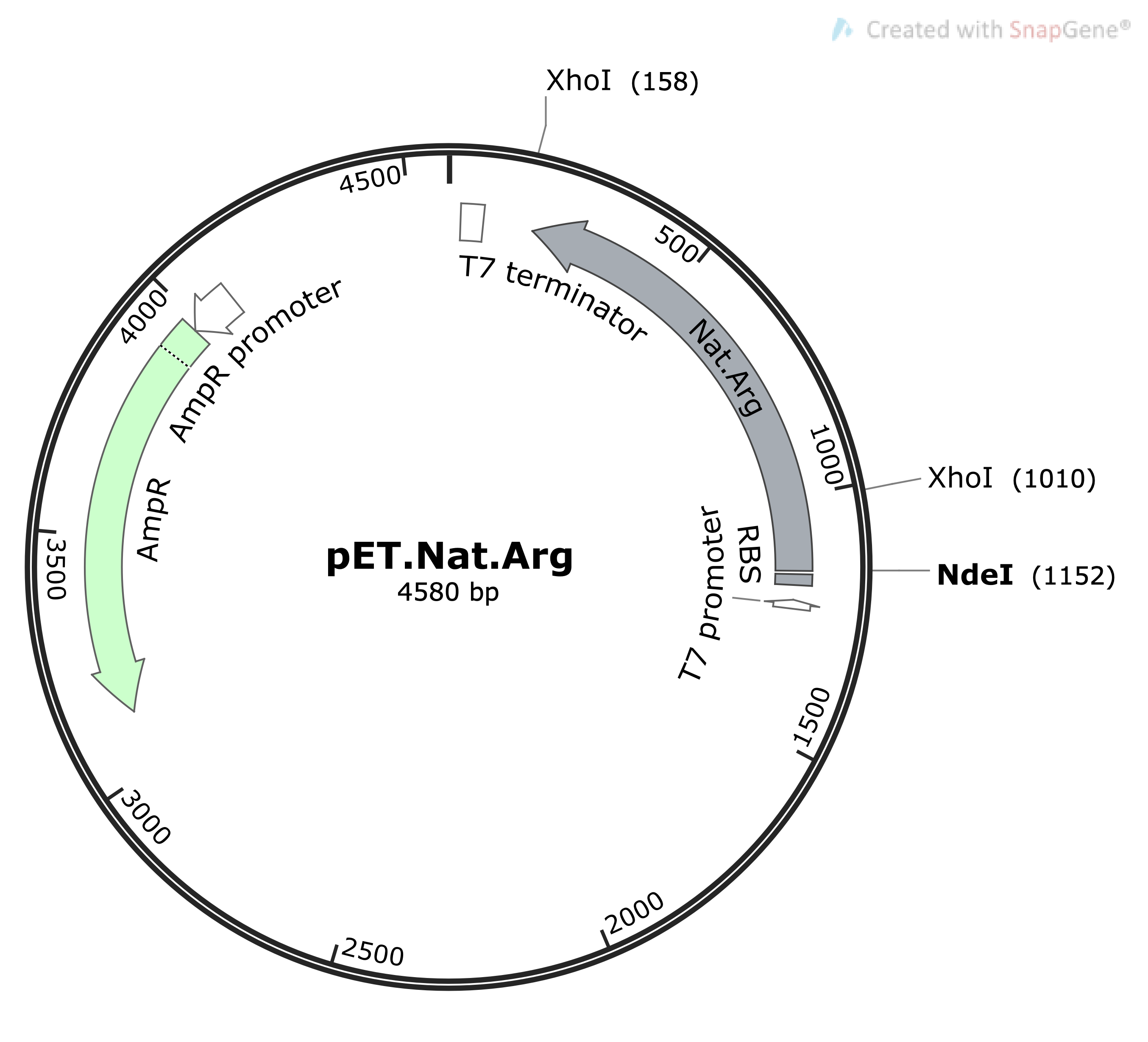

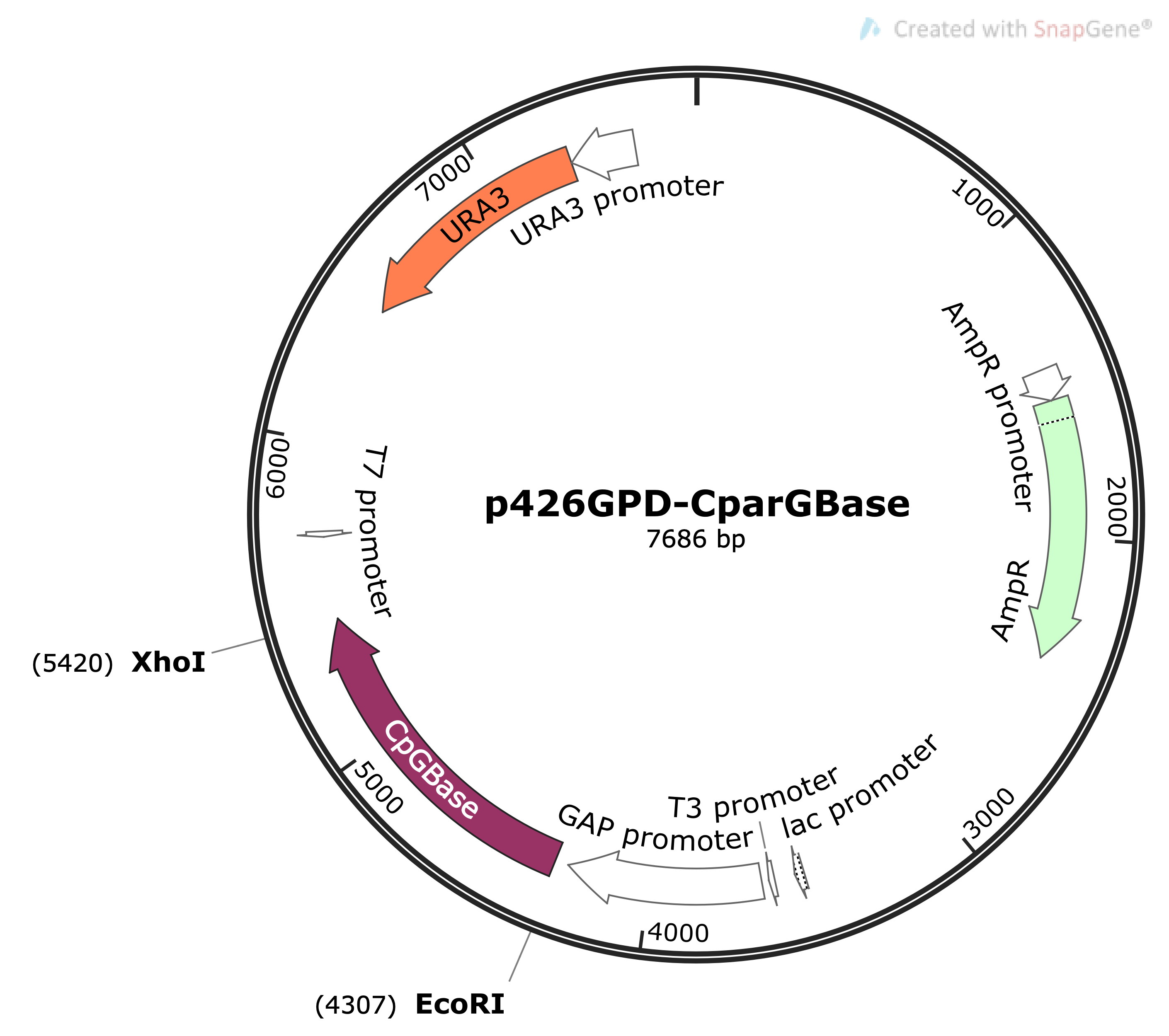

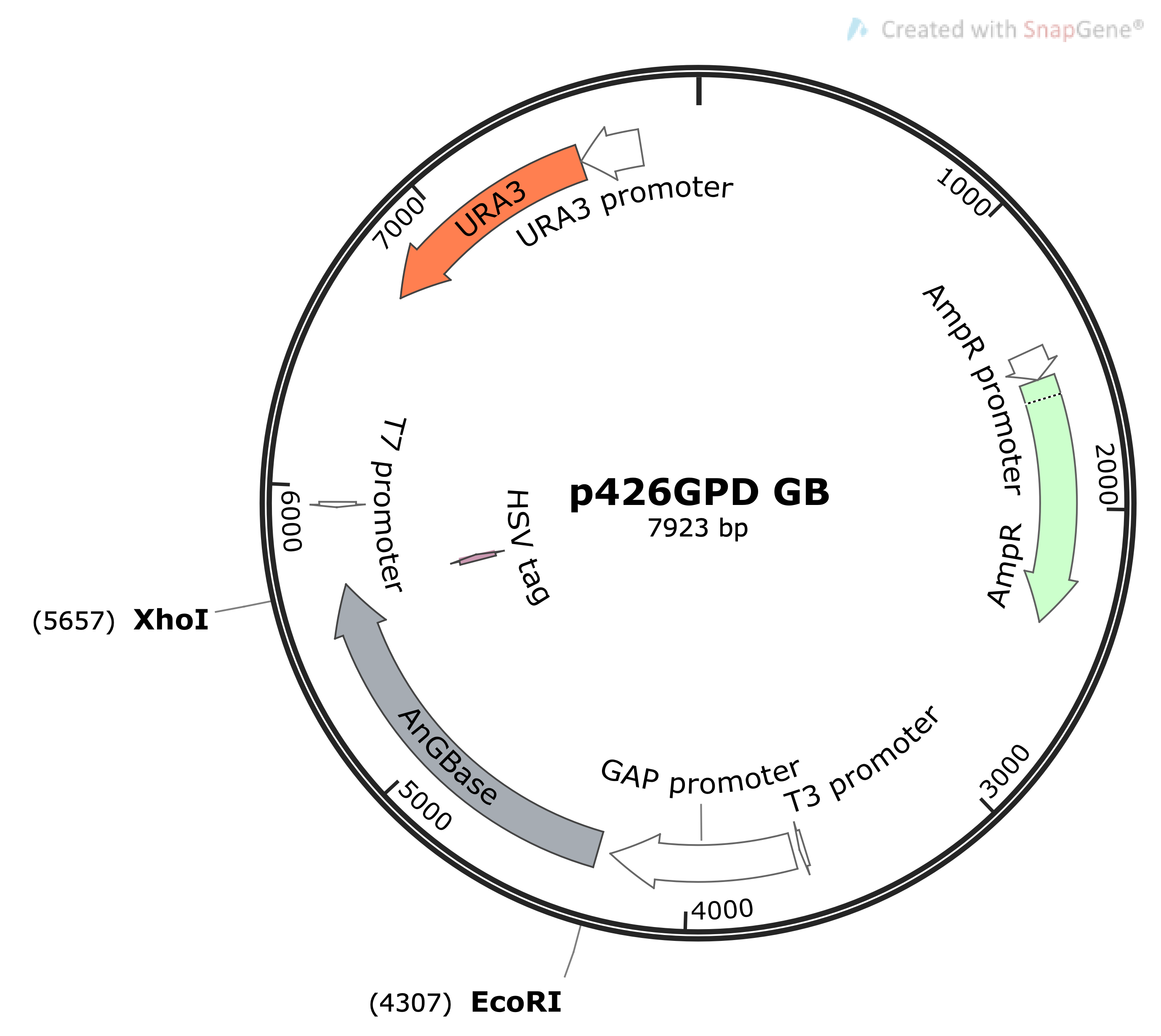

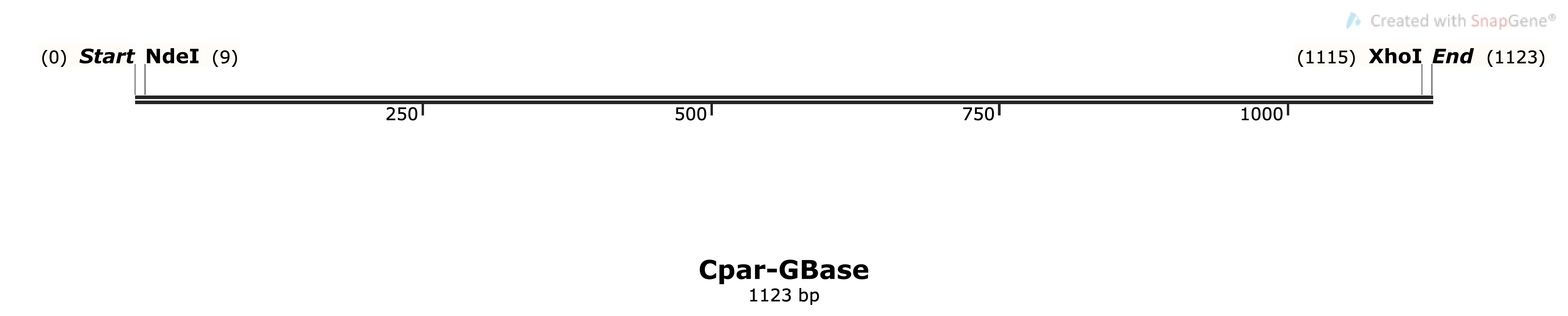

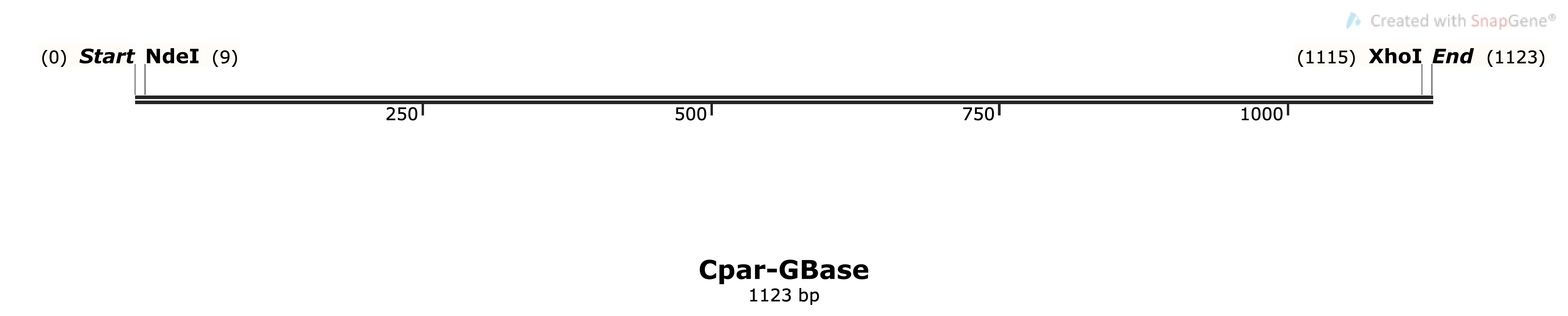

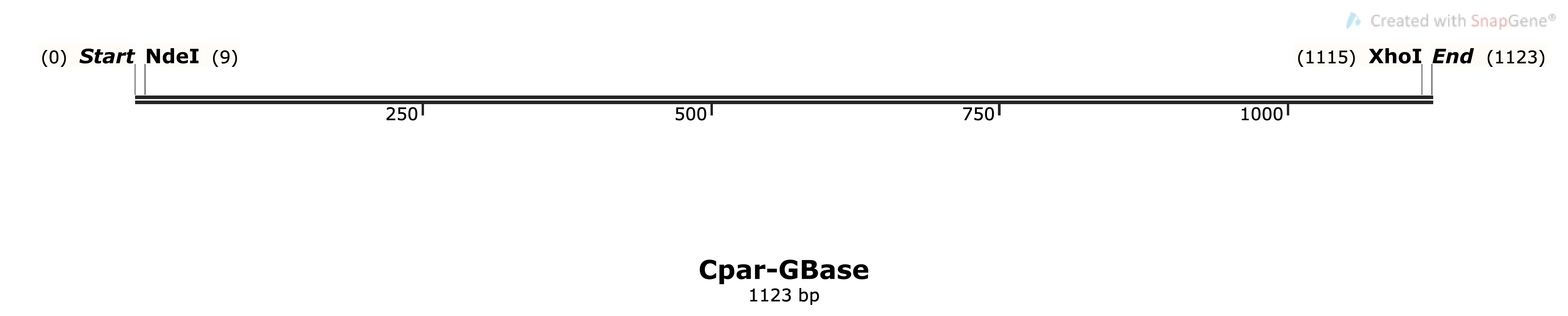

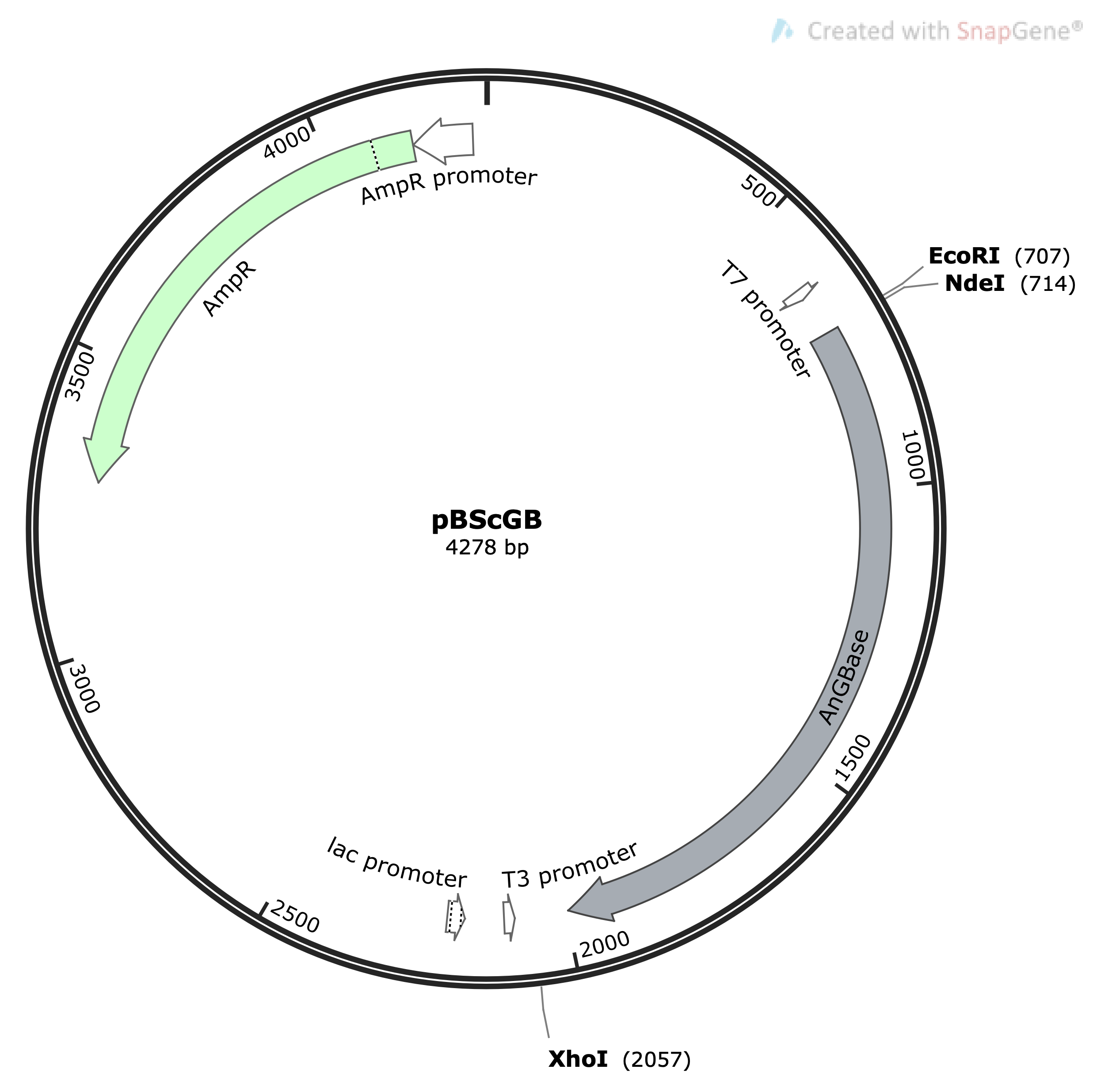

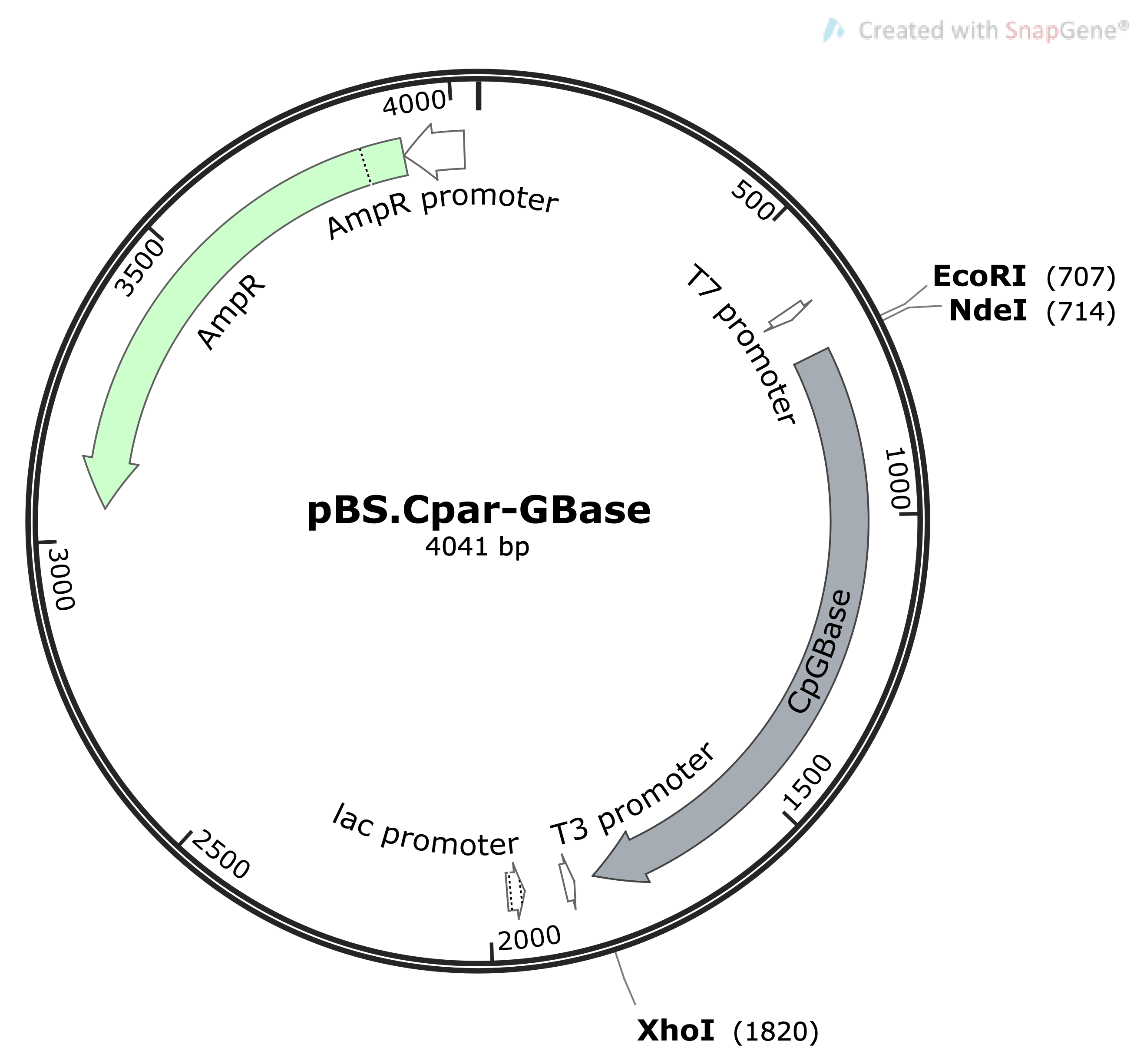


*Nde*I

*Xho*I

*Nde*I

*Xho*I

*EcoR*I

*Xho*I

**Supplementary Figure S2. Construction of pCB-PcitACpar for CpGBase expression in *A. niger*.** Strategy for the construction of pCB-Aga-Cpar and pCB-PcitA Cpar plasmids. The gel image represents the restriction digestion of the final plasmid analyzed by agarose gel electrophoresis. Lane 1- 1 kb DNA ladder, Lane 2- *Nde*I-*Not*I digest, Lane 3-*Eco*RV-*Hind*III digest, Lane 4- *Pvu*II diges, Lane 5- 2 kb DNA ladder, Lane 6- *Nde*I-*Not*I digest, Lane- 7 *Xba*I-*Not*I digest, Lane -8 *Pvu*II digest.


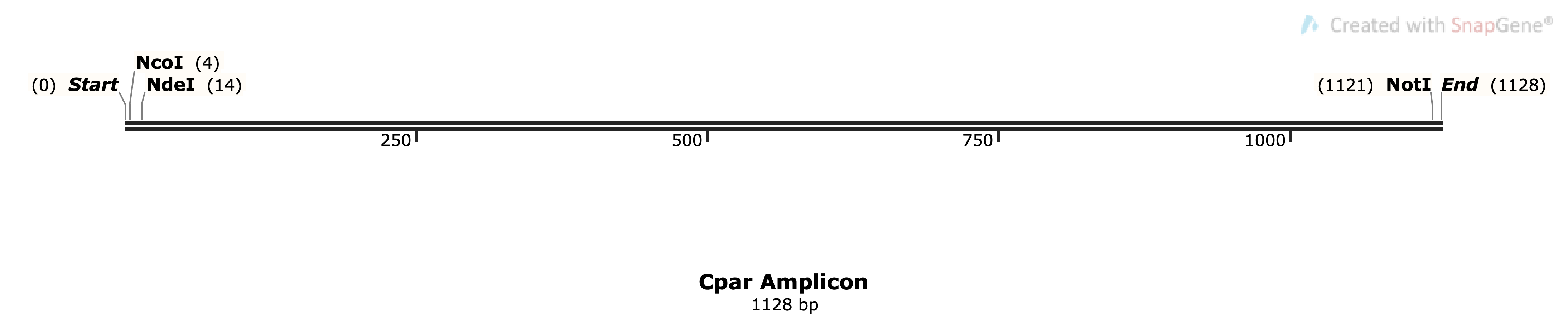

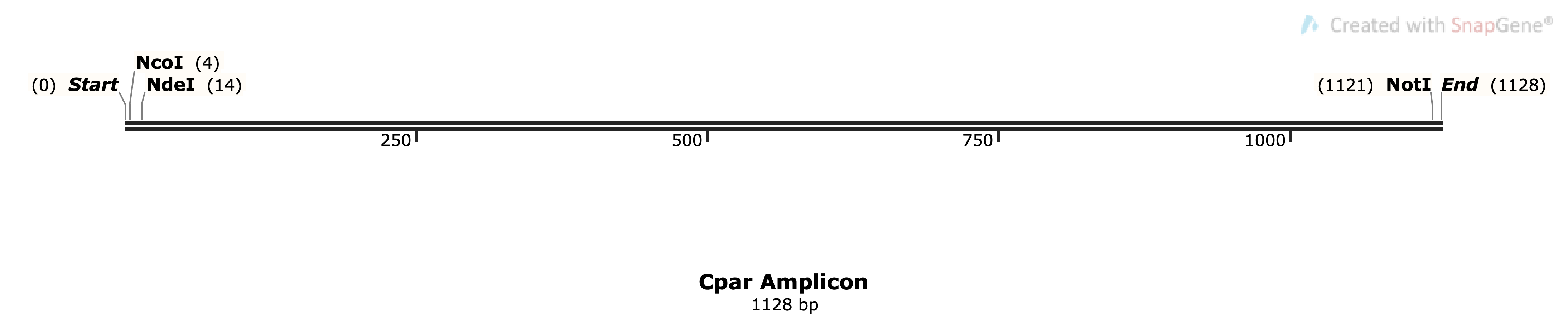

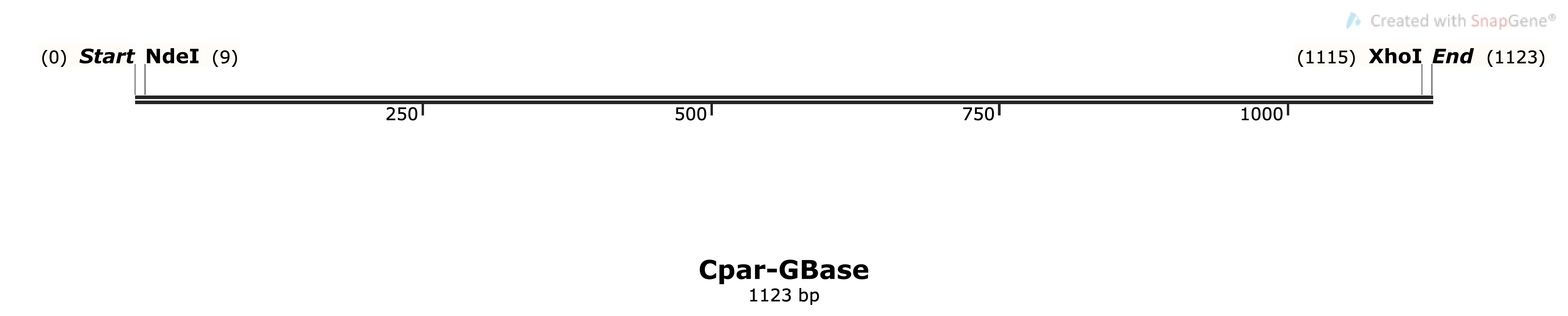

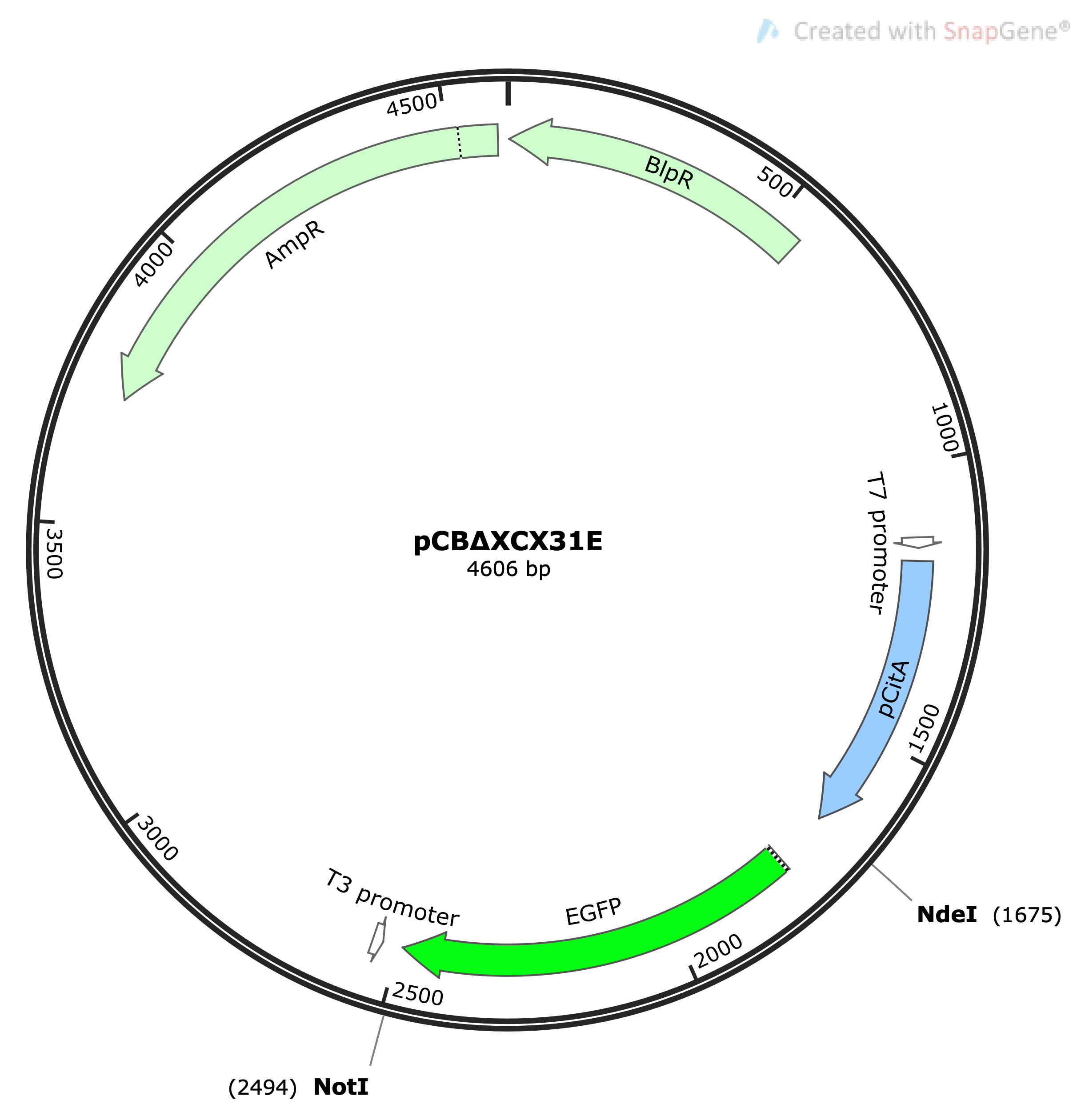

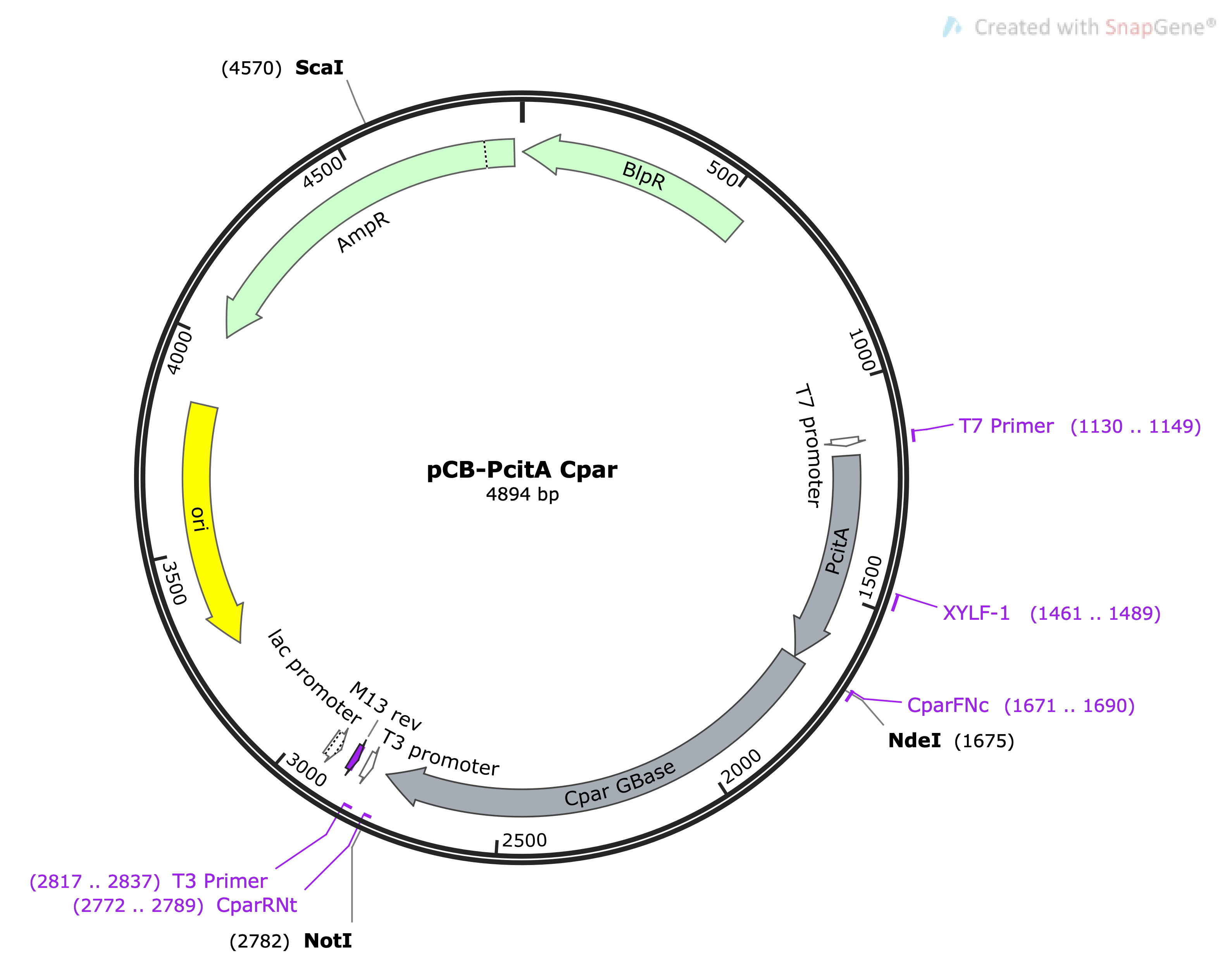

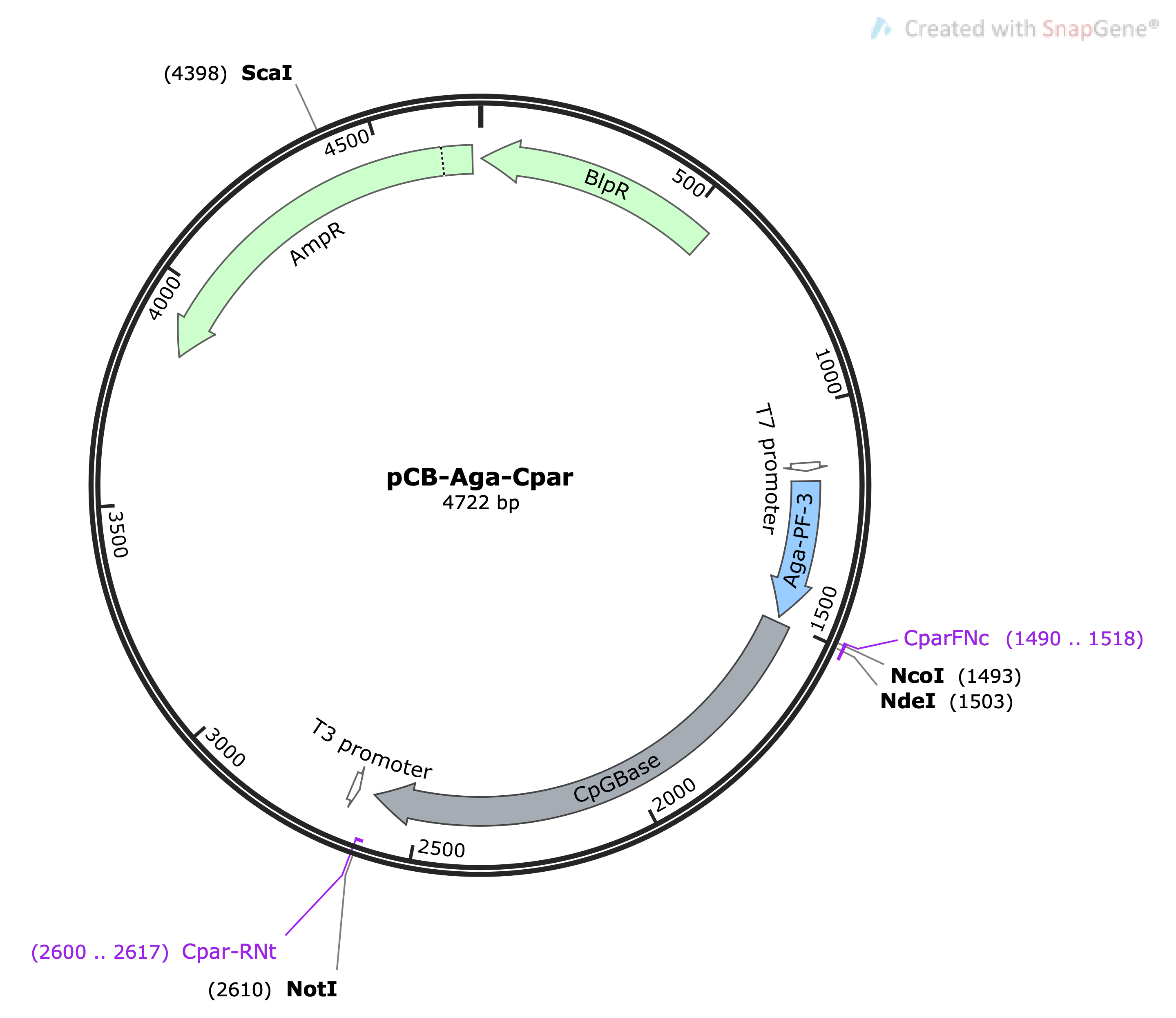


*Nco*I

*Not*I

*Nde*I

*Not*I


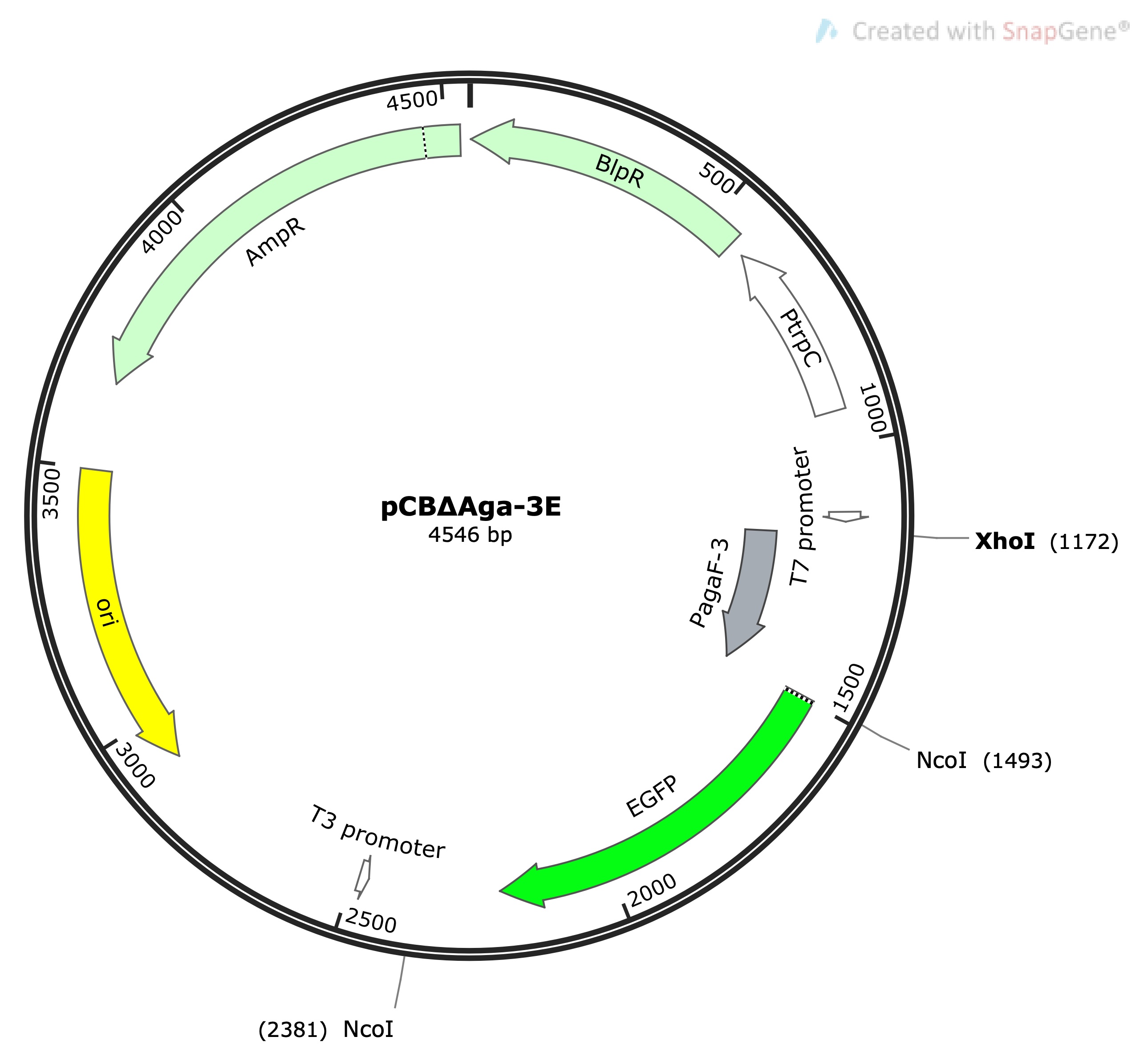

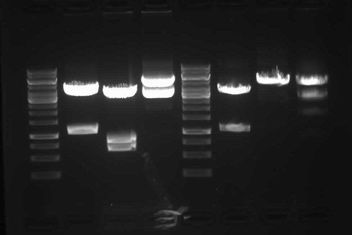


1 2 3 4 5 6 7 8

**Supplementary Information S1**

**The assay method developed to estimate 4-guianidinobutyrase (GBase) using DMAB reagent**

Archibald’s method for measuring urea has a detection limit of 0.5 mM. The method also requires strong acids and boiling to develop color, which is time-consuming. To overcome these shortcomings, the present DMAB (*p*-dimethylaminobenzaldehyde) method was adopted to measure urea formation in the GBase reaction.

A 4% (w/v) solution of DMAB in either absolute ethanol or acetonitrile and 4% (v/v) of sulfuric acid reagent was made and kept at 4 °C. The reagent was diluted in distilled water (1:2 vol) before adding the reagent to stop the reaction. The tubes, after the addition of the DMAB reagent, were incubated for 10 min at room temperature and absorbance was measured at 420 nm.

Urea (mM)

A_420_

**Supplementary Figure S3. Urea standard curve using the DMAB method (R^2^ = 0.9993).** Different concentrations of urea (0.1 mM to 8.0 mM in distilled water) were to build the standard curve.

The DMAB method was standardized for pharmaceutical samples (Knorst et al., 1997). The method is simple, does not require strong acids and takes 10 min to develop color. Therefore, this method was optimize for the GBase assay. The results show (Supplementary Figure S3) that the DMAB in the ethanol as a reagent, is more sensitive at lower urea concentrations. This DMAB reagent was chosen for further standardization.


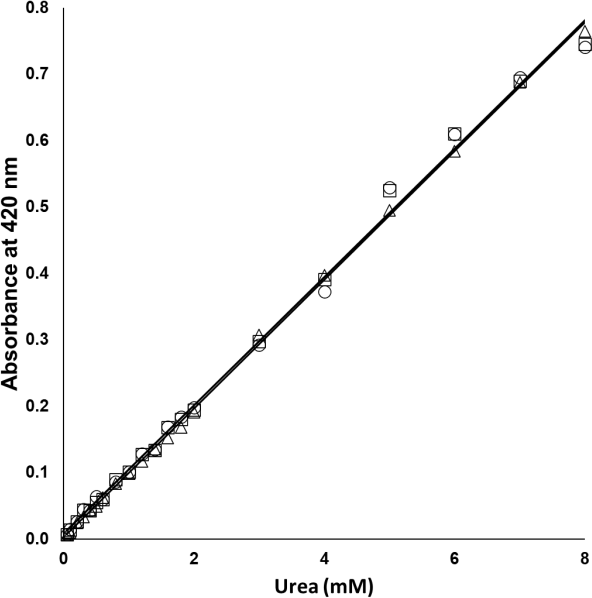
Archibald’s method to develop color for the GBase assay (0.5 mL reaction, of which two 200 µL aliquotes were used as replicates) employed a 200 µL reaction mixture sample (along with 4 mL acid reagent + 200 µL color reagent). However, the DMAB assay (Knorst et al., 1997) was standardized for a reaction volume of 2.5 mL and subsequently was reduced to 1.25 mL (0.25 mL reagent + 1 mL sample) and 0.625 mL (0.125 mL reagent + 0.5 mL sample) to increase the sensitivity of the assay (Supplementary Figure S4).

**Supplementary Figure S4. DMAB assay standardized to measure absorbance using different reaction volumes.** Standard urea solutions were used to develop color from an assay reaction volume of 2.5 mL (∆, R^2^ = 0.9993), 1.25 mL (○, R^2^ = 0.9957) and 0.625 mL (□, R^2^ = 0.9959).


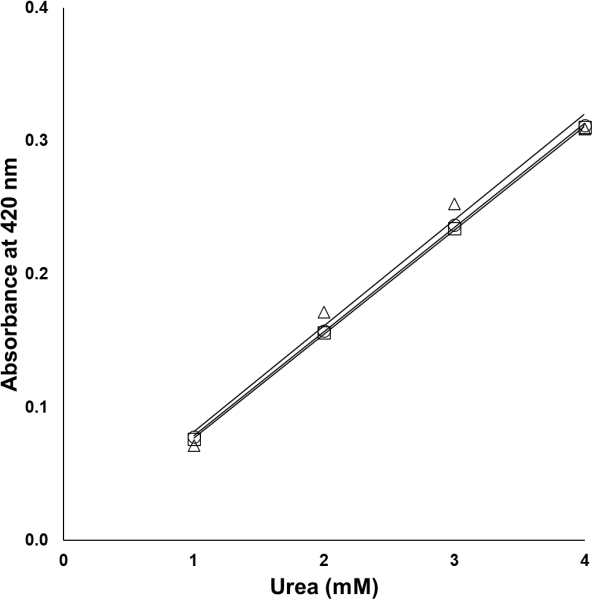
The phosphate buffer (100 mM) used in the GBase assay forms a precipitate with DMAB reagent – due to ethanol and phosphate. The phosphate buffer concentration was reduced to 20 mM (still maintains the reaction pH at 7.5) in a total reaction volume of 1.25 mL (0.25 mL reagent + 0.5 mL reaction + 0.5 mL distilled water). Nevertheless, reaction mixtures (after DMAB reagent addition) were centrifuged to remove the precipitate, if any. The results obtained (Supplementary Figure S5) showed no precipitation of phosphate buffer.

**Supplementary Figure S5. Comparison of urea standard curves to account for phosphate buffer precipitation.** Standard urea solutions were used to develop color from an assay reaction volume of 1.25 mL, before centrifugation (○, R^2^ = 0.9998) and after centrifugation (□, R^2^ = 0.9999). A standard curve for urea in plain water (∆, R^2^ = 0.9991) is also shown.

The stability of the color formed upon DMAB reaction with urea was monitored. The color was stable for up to 60 min (Supplementary Figure S6).


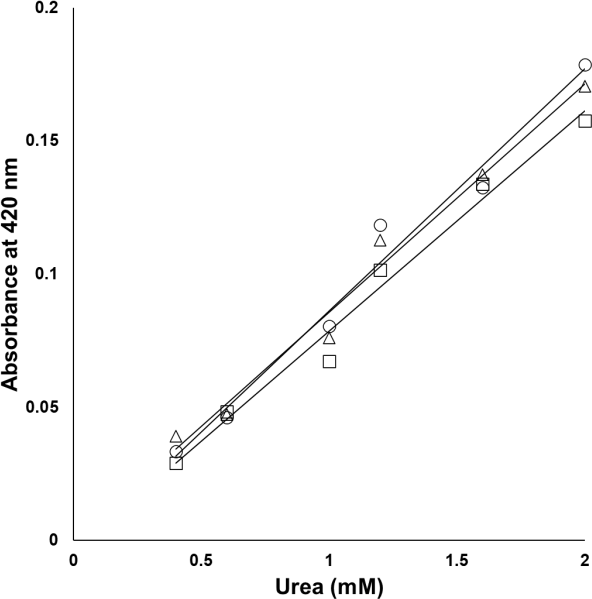


#### Supplementary Figure S6. Stability of colored product formed in DMAB reaction with urea. The urea standard curve generated after the DMAB reaction in the presence of 20 mM phosphate buffer and GB (25 mM) was monitored for color stability after 10 min (∆, R^2^ = 0.9876), 30 min (○, R^2^ = 0.9877) and 60 min (□, R^2^ = 0.9869).

**Supplementary Information S2**

GBase activity in cell-free extract using DMAB assay: The cell-free extract (from *C. parapsilosis*) was assayed for GBase activity using the DMAB method developed above. There was a high background colour in the presence of elevated concentrations of protein (Supplementary Table S1). This assay cannot be used for cell extract samples with high protein concentrations.

**Supplementary Table S1 GBase activity in cell free extracts using DMAB assay**

| **Protein** | **Zero min Blank** | **After 15 min reaction** | **Difference** |
| --- | --- | --- | --- |
| 10 µg | 0.0323 | 0.0723 | 0.0400 |
| 20 µg | 0.1076 | 0.1610 | 0.0534 |
| 40 µg | 0.2130 | 0.3199 | 0.1070 |

### DMAB assay with enriched GBase enzyme: To avoid the high background values, the enriched GBase enzyme was used for substrate saturation studies. A K_M_ of 2.7 ±0.3 mM for GB was obtained for AnGBase earlier, using the HPLC method (Saragadam *et al.,* 2019). A GB substrate saturation (1 to 20 mM) experiment with the standardized DMAB method is shown in Supplementary Figure S7. The The results obtained are shown in Supplementary Figure S 2.5. A K_M_ of 2.8±0.3 obtained using the DMAB method compares well with the earlier results.

####
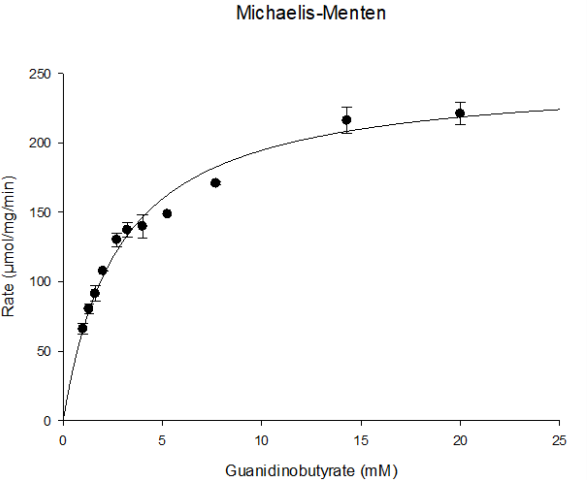


#### Supplementary Figure S7. An GBase substrate saturation monitored using the DMAB method.

**Supplementary Figure S8.** The ClustalW multiple alignment of 4-guanidinobutyrase (GBase) protein sequences from *Aspergillus niger* NCIM 565 (AnGBase, Accession No. AHL44994) and *Candida parapsilosis* NCIM3689 (CpGBase Accession No. OK067409). These sequences were used for building homology models of AnGBase and CpGBase.

**References:**

Archibald, R. M. (1944). Determination of citrulline and allantoin and demonstration of citrulline in blood plasma. *J. Biol. Chem*. 156, 121.

Boyde, T. R. & Rahmatullah, M. (1980). Optimization of conditions for the colorimetric determination of citrulline, using diacetyl monoxime. *Anal. Biochem.* 107, 424-431.

Knorst, M. T., Neubert, R., & Wohlrab, W. (1997). Analytical methods for measuring urea in pharmaceutical formulations. *Journal of Pharmaceutical and Biomedical Analysis*, *15*(11), 1627–1632. <https://doi.org/10.1016/s0731-7085(96)01978-4>
